# Targeting the Eye: RNA-Based Therapies, Interferences, and Delivery Strategies

**DOI:** 10.3390/pharmaceutics17101326

**Published:** 2025-10-13

**Authors:** Mohammed S. Abdel-Raziq Hassan, Cheng Zhong, Fatma Hassan, S. Kevin Li

**Affiliations:** 1 Department of Chemistry, College of Arts and Sciences, University of Cincinnati, Cincinnati, OH 45221, USA; 2 Division of Pharmaceutical Sciences, James L Winkle College of Pharmacy, University of Cincinnati, Cincinnati, OH 45267, USA

**Keywords:** RNA nanotechnology, RNA aptamer, siRNA, miRNA, shRNA, antisense oligonucleotides, eye, posterior eye disease

## Abstract

Recent advances in molecular biology have led to the development of RNA-based therapeutics, offering significant promise for treating various eye diseases. Current RNA therapeutics include RNA aptamers, antisense oligonucleotides (ASOs), small interfering RNA (siRNA), and messenger RNA (mRNA) that can target specific genetic and molecular pathways involved in eye disorders. In addition to their potential in therapy, RNA technologies have also provided tools for mechanistic studies to improve the understanding of eye diseases, expanding the possibilities of RNA-based treatments. Despite the utility of RNA in studying eye disease mechanisms and its potential in disease treatment, only a few RNA-based therapies have been approved for posterior eye diseases. This paper reviews RNA interference and related ocular delivery and posterior eye diseases, focusing on the use of RNA aptamers, siRNA, short hairpin RNA (shRNA), and microRNA (miRNA). Approaches using RNA to advance our understanding of eye diseases and disease treatments, particularly in the posterior segment of the eye, are discussed. It is concluded that RNA therapeutics offer a novel approach to treating a variety of eye diseases by targeting their molecular causes. siRNA, shRNA, miRNA, and ASO can directly silence disease-driving genes, while RNA aptamers bind to specific targets. Although many RNA-based therapies are still in experimental stages, they hold promise for conditions such as age-related macular degeneration (AMD), diabetic macular edema (DME), glaucoma, and inherited retinal disorders. Effective delivery methods and long-term safety are key challenges that need to be addressed for these treatments to become widely available.

## 1. Introduction

Recent advances in molecular biology have led to the development of RNA-based therapeutics, which hold significant promise for treating a variety of eye diseases [1,2,3]. RNA therapeutics encompass a range of approaches including RNA aptamers, antisense oligonucleotides (ASO), small interfering RNA (siRNA), and messenger RNA (mRNA) therapies [4,5]. These strategies target specific genetic and molecular pathways involved in eye disorders, offering potential for precision medicine. Over the past few decades, RNA technologies have also provided tools to improve the understanding of eye diseases. Basic and preclinical research studies involving RNA aptamers, siRNA, short hairpin RNA (shRNA), and microRNA (miRNA) have emerged to investigate the mechanisms of eye diseases and expand the possibilities of using RNA-based approaches to improve eye disease treatment.

There are only a few RNA-based therapies approved for diseases in the posterior segment of the eye. FDA-approved RNA-based therapies for posterior eye diseases include Vitravene (fomivirsen), an antisense oligonucleotide for cytomegalovirus retinitis (CMV) in 1998; Macugen (pegaptanib), an aptamer targeting vascular endothelial growth factor (VEGF) protein for wet age-related macular degeneration (AMD) in 2004; and Izervay (avacincaptad pegol), an aptamer targeting complement protein C5 for geographic atrophy (GA) secondary to AMD in 2023. Both Vitravene and Macugen are discontinued and no longer available in the United States. Vitravene was withdrawn due to low demand and Macugen was discontinued after more effective anti-VEGF therapies (e.g., anti-VEGF antibody, antibody fragment, and fusion protein) became available. To our knowledge, there is no approved RNA-based therapy with gene delivery or RNA interference (RNAi) that silences gene expression for posterior eye diseases. A gene therapy (Luxturna) was approved in 2017 by the FDA for RPE65 mutation-associated retinal dystrophy in the treatment of Leber Congenital Amaurosis (LCA) by subretinal injection, but it is not based on RNA technologies. Luxturna (voretigene neparvovec-rzyl) is an adeno-associated virus modified to express the human RPE65 gene, and the virus vector-based gene therapy is derived using recombinant DNA techniques.

This paper presents a concise overview of ocular anatomy, posterior segment eye diseases, ocular drug delivery strategies, and RNA-based technologies. Emphasis is placed on the application of RNA modalities—siRNA, miRNA, shRNA, RNA aptamers, and ASO—in both the investigation and treatment of diseases affecting the posterior segment of the eye. The review first explores how RNA interference tools have been employed to elucidate disease mechanisms, thereby advancing our understanding of ocular pathophysiology. It then highlights the advances made in RNA-based therapeutics, showcasing examples with clinical potential. Collectively, these RNA approaches represent a promising development in targeted and transformative treatments for posterior eye diseases.

## 2. Posterior Eye Diseases and Ocular Delivery

### 2.1. Anatomy of the Eye

**Figure 1:**
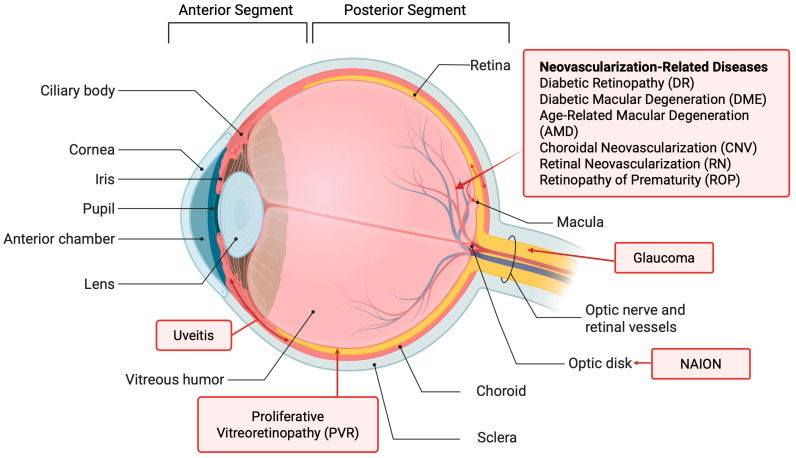
Anatomy of the eye and eye diseases. Created in BioRender. https://BioRender.com/n5t667w (accessed on 19 September 2025).

Before the review of posterior eye diseases, this section provides a brief overview of eye anatomy. The human eye is a slightly asymmetrical globe with a diameter of around 23 mm. The eye can generally be divided into the anterior and posterior segments (Figure 1). The anterior segment of the eye occupies the front one-third of the eye, including the cornea, pupil, iris, ciliary body, anterior chamber, and lens. The aqueous humor is produced at the ciliary body and fills the anterior segment of the eye. The posterior segment occupies two-thirds of the back of the eye, including the vitreous, choroid, retina, macula, and optic nerve. The retinal pigment epithelium (RPE) is between the retina and choroid and has a major role in maintaining the function of the retina. The vitreous is filled with vitreous humor, a clear gelatinous fluid. The light-sensing cells in the retina convert light into signals for vision. Many posterior eye diseases are related to the retina and its surrounding tissues. The unique structures of the eye have posed a challenge to drug delivery to the posterior segment of the eye (see Section 2.3).

### 2.2. Posterior Eye Diseases

Diseases in the posterior segment of the eye account for the majority of vision loss in the United States [6]. Patients suffering from posterior eye diseases are in need of therapies that are both effective and non-burdening. The posterior eye diseases covered in this review are illustrated in Figure 1 and summarized in Table 1. These diseases have been previously reported in the studies and/or treatments using RNA (see Section 3, Section 4 and Section 5). They are the focus of this review.

DR is a condition prevalent in diabetic patients. The early stage of DR is non-proliferative (NPDR). Severe NPDR could progress to proliferative DR (PDR). In PDR, abnormal growth of new blood vessels takes place at the back of the eye. The formation of new blood vessels and the leaking of blood vessels in the retina lead to vision loss. DME is a vision-threatening complication for diabetic patients and is characterized by the accumulation of fluid, swelling, and thickening of the macula, resulting in vision loss.

AMD is a vision-threatening disease related to the deterioration of the macula. AMD occurs in two forms: dry and wet AMD. Dry AMD is characterized by the growth of drusen and the atrophy of the RPE that slowly affects the central vision. In wet AMD, abnormal blood vessel growth occurs, which often leads to blood leakage and eventually damages the macula.

In the treatment of neovascularization in the posterior eye, VEGF is a major target because of its roles in angiogenesis and vascular permeability [7,8]. This approach has led to the successful development of anti-VEGF agents for DR, DME, and AMD. For example, ranibizumab (Lucentis), aflibercept (Eylea), brolucizumab (Beovu), and bevacizumab (Avastin, off-label use) are anti-VEGF proteins for these eye diseases [9]. Pegaptanib sodium (Macugen) is an anti-VEGF RNA aptamer for wet AMD [7], although it is seldom used now due to its inferior treatment outcome. In addition to VEGF, Angiopoietin-2 (Ang2), which can enhance the angiogenic effects of VEGF [10,11], has also been antagonized in therapeutic strategies [12,13,14]. Faricimab (Vabysmo), a bispecific antibody that binds to both VEGF-A and Ang2, was recently approved for the treatment of patients with neovascular AMD and DME [15].

ROP is a retinal vascular disease in premature infants and is related to abnormal blood vessels in the retina. ROP can be generally categorized into five different stages. Patients with mild conditions of ROP (Stages 1 to 2) can recover without treatment. Some patients in Stage 3 need treatment to protect their vision. Patients with ROP in Stages 4 and 5 require treatment to prevent blindness. Current treatments of ROP include anti-VEGF agents, laser, and eye surgery.

PVR is a condition due to anatomic disruption and damage to the retina, resulting in scar tissue and inflammation. PVR is characterized by the proliferation of membranes within the retina and vitreous cavity and the resulting vision loss. There is no approved drug for PVR, and the primary treatment is retinal surgery.

Glaucoma is characterized by the loss of peripheral vision, followed by the loss of central vision and blindness. The disease is related to the loss of retinal ganglion cells and optic neuropathy. Elevated intraocular pressure (IOP) is the major risk factor of glaucoma. For example, elevated IOP can damage the cells related to and around the optic nerve. Current treatment of glaucoma is by IOP-controlling agents such as prostaglandin analogs, beta blockers, carbonic anhydrase inhibitors, and a rho-kinase (ROCK) inhibitor. There is no RNA-based therapy approved for the treatment of glaucoma as of now.

Uveitis is the inflammation of the uvea, which consists of the iris, ciliary body, and choroid. Uveitis can be classified as anterior uveitis, intermediate uveitis, and posterior uveitis, depending on the site of inflammation. Posterior uveitis is a form of inflammation affecting the retina and choroid, leading to vision loss. The standard treatment of posterior uveitis is intravitreal administration of anti-inflammatory agents such as corticosteroids.

RP is a degenerative eye disease caused by genetic defects. The disease is related to the abnormalities of the photoreceptors and the slow degeneration of the retina. RP is an inherited degenerative eye disease that leads to night blindness and progressive constriction of the visual field. Currently, no approved drug can effectively treat RP.

NAION is the restriction of blood flow to the optic nerve. NAION is a form of anterior ischemic optic neuropathy (AION) or the ischemic optic neuropathy of the optic nerve head (i.e., optic disc) in the posterior segment of the eye. When the optic nerve is deprived of oxygen due to the loss of blood supply, cell death and nerve damage occur, leading to vision loss. Currently, no effective treatment is available for NAION.

### 2.3. Ocular Drug Delivery

The major routes of ocular drug delivery include topical, systemic, periocular, and intravitreal administration [16,17,18,19]. The topical route is the most common for drug delivery to the anterior segment of the eye, but it is not effective for the posterior segment. Topical delivery is limited by the permeability of the cornea and precorneal clearance from nasolacrimal drainage and tear fluid turnover. For example, drugs in conventional eye drops can only remain on the eye for a short period of time [20,21] and only a small fraction (< 5%) of the applied dose can be absorbed into the eye through the cornea [22]. With the distance between the anterior and posterior segments of the eye, aqueous humor flow, and turnover rate in the anterior chamber, the topical route is considered ineffective for posterior eye delivery and disease treatments.

Systemic administration can deliver drugs to the posterior segment of the eye via the systemic circulation (e.g., by oral administration). However, the systemic route is limited by the blood-retina barrier (BRB). With the adverse effects related to systemic exposure and the frequent administration required to reach therapeutic levels in the eye, the systemic route is generally not preferred for posterior eye diseases.

Subconjunctival, sub-Tenon’s, retrobulbar, peribulbar, and posterior juxtascleral routes are examples of periocular administration. Periocular administration can take advantage of the large surface area and high permeability of the sclera. For example, the subconjunctival route delivers the therapeutics to the subconjunctival pocket under the conjunctiva to avoid the conjunctival-cornea barrier for direct access to the sclera in transscleral delivery. Despite that, the need to overcome several static and dynamic barriers (tissues and clearance, respectively) to reach the target sites in the choroid, RPE, and/or the retina has limited the effectiveness of periocular administration. 

The most common route of administration to deliver therapeutics to the posterior segment of the eye is intravitreal administration, e.g., the direct injection of therapeutics into the vitreous via the pars plana [23]. The current anti-VEGF therapies are administered by intravitreal injection. By avoiding the ocular delivery barriers via direct injection to the vitreous, this route can provide the desired concentration of therapeutics in the vitreous and retina, which cannot be achieved by topical or systemic administration. However, intravitreal administration is invasive, and frequent injections are required for chronic eye diseases. Repeated injections can increase the risks of adverse effects such as increased IOP, retinal detachment, vitreous hemorrhage, inflammation, endophthalmitis, and cataract [24,25]. For RNA therapeutics and gene delivery to the posterior eye segment, subretinal and suprachoroidal administration are sometimes preferred. Unlike intravitreal administration, which usually targets the outer retina such as the retinal ganglion cells, the subretinal and suprachoroidal routes can be utilized to target the inner structure of the retina with higher transduction efficiency. However, the subretinal and suprachoroidal routes are considered more invasive. The RNA and RNA therapeutics covered in this review were mostly delivered into the eye by the intravitreal route and some by the subretinal and suprachoroidal routes (see Section 3 and Section 4). Table 2 summarizes the common modes of ocular drug delivery.

### 2.4. RNA Delivery

RNA is vulnerable to RNases and phosphatases in the body. It is relatively unstable due to the plethora of ribonucleases in serum and cells. Therefore, the current RNA-based therapies require high concentrations to achieve therapeutic effects. In addition, RNA can be unstable at ultra-low concentrations after being administered and diluted in the body. To be delivered to the target sites in the cells, RNA-based therapeutic agents are required to pass through the extracellular matrix and across the cell membrane via endocytosis in a non-toxic manner [26,27]. Chemical modifications, such as the modifications of 2′ sugar at the RNA ribose including 2′-fluoro (F) and 2′-methoxyethyl (MOE) and phosphorothioate (PS) modifications, are promising strategies to improve the stability of RNA-based therapeutics and reduce immunogenicity without reducing activity [26,28]. In addition, a delivery vehicle to efficiently transport RNA-based agents to their targets through the extracellular and intracellular barriers can be an effective strategy [26,29]. Nanotechnology provides advantages and potential solutions to the challenges of RNA delivery. Physical enhancement methods such as those involving poration (microneedle), ultrasound (sonophoresis), or electric field (electroporation and/or iontophoresis) can also improve ocular drug delivery and provide advantages over traditional passive drug delivery, but they will not be discussed further in this review.

### 2.5. Particulate Systems for Ocular Delivery

Particulate drug delivery systems, such as microparticles and nanoparticles, are attractive drug delivery platforms to improve ocular delivery [30]. Recent progress in biomaterials and nanotechnology has led to advances in biodegradable microparticles and nanoparticles. Microparticles are common in ocular drug delivery and can offer longer retention and higher drug-loading capacity than nanoparticles. However, microparticles usually cannot penetrate membranes to provide targeted delivery and therefore will not be discussed further in this review.

Nanoparticles have been widely studied and employed in the field of ocular drug delivery [22]. The advantages of these carrier systems include sustained drug release, targeted delivery, potential to overcome ocular delivery barriers, and enhanced stability of the drug content [31]. These advantages can improve drug bioavailability at the sites of action for therapeutic effects in the eye. Examples of nanosystems in ocular delivery and therapies are (a) polymeric nanoparticles such as poly(lactic-co-glycolic) acid (PLGA) and chitosan-sodium alginate nanoparticles [32,33,34,35], (b) lipid-based nanoparticles such as liposomes, solid lipid nanoparticles (SLN), nanoemulsion, and micelles [36,37,38,39,40], (c) inorganic nanoparticles such as gold nanoparticles [41,42,43], and (d) biological nanoparticles such as exosomes and nucleic acid-based nanoparticles [44,45,46]. Nanosystems can be employed for RNA therapeutics [47,48], which will be discussed in Section 2.6.

### 2.6. Nanosystems for Ocular RNA Therapeutics

As discussed in Section 2.4, RNA-based therapeutics have encountered enzymatic degradation, low retention, and off-target effects, limiting their clinical applications in ocular therapies [48,49]. Chemical modifications of RNA to enhance its stability from nuclease degradation can promote its biological activity and lower its immunogenicity. Besides chemical modifications, encapsulating RNA in nanoparticles as carriers can improve the stability and bioavailability of RNA therapeutics by preventing their degradation. As protective materials, the nanocarriers can also reduce the immunogenicity of RNA therapeutics, resulting in improved efficacy. In addition, nanocarriers can enhance the retention of RNA therapeutics in the eye, provide precise targeting ability, improve cellular penetration and cytoplasmic release, and minimize toxicity to eye tissues.

**Figure 2:**
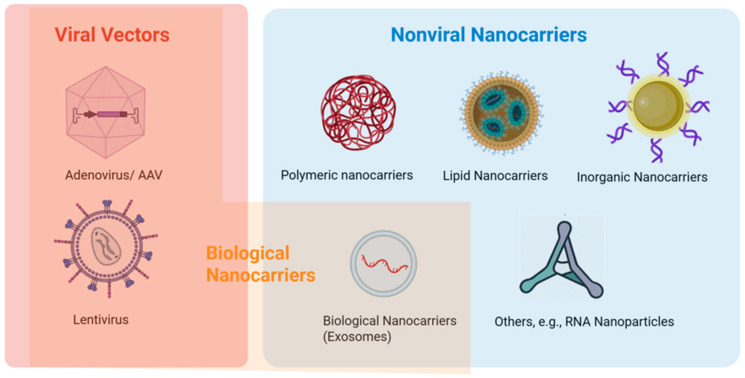
Nanosystems for ocular therapeutics. Created in BioRender. https://BioRender.com/fci6958 (accessed on 19 September 2025).

There are two main types of nanocarriers: viral vectors and nonviral nanocarriers (Figure 2). For viral vectors, the cellular uptake is usually through endocytosis and then endosomal escape by membrane fusion between the viral vector and endosomal membranes, releasing the viral contents into the cytoplasm. Nonviral nanocarriers have distinct mechanisms for cellular uptake and endosomal escape. The carriers can be internalized through cellular uptake by endocytosis and endosomal escape through rupturing the endosome or fusion of the nanocarrier membrane with the endosomal membrane [49,50]. In general, nanocarriers/nanoparticles can be categorized into polymer-based, lipid-based, inorganic, biological, their hybrid, and other carriers [48,49]. Many studies covered in the present review employed nanosystems to deliver siRNA, microRNA, shRNA, and RNA aptamers in cell culture in vitro and animals in vivo (see Section 3 and Section 4).

#### 2.6.1. Polymeric Nanocarriers

Polymeric nanocarriers are made of natural or synthetic materials. These nanoparticles show great potential for ocular delivery applications due to their tunable features such as surface modification. Examples of polymeric nanocarriers are trimethyl chitosan-HA nanopolyplexes [51], poly(lactic-co-glycolic acid) (PLGA) nanoparticles grafted with the short peptide arginine-glycine-aspartate [52], and core–shell multilayer nanosystem [53]. Improving encapsulation efficiency, ensuring safety, and minimizing off-target effects are the key factors in nanocarrier development. Despite the ability of polymeric nanocarriers to control the particle and surface properties for RNA delivery, polymeric nanocarriers still have obstacles for effective delivery due to the limited biodegradability and potential toxicity.

#### 2.6.2. Lipid Nanocarriers

Lipid nanoparticles (LNPs) can be utilized as a nonviral platform for mRNA delivery. A recent example is the coronavirus disease 2019 (COVID-19) vaccine [54,55]. A number of recent reviews have covered this topic [56]. For ocular drug delivery, LNPs are promising carriers due to their simple formulation, use of Generally Recognized as Safe (GRAS) excipients, and scalable production. Despite these advantages, it has been reported that LNP-mediated mRNA delivery cannot effectively penetrate the neural retina regardless of subretinal or intravitreal administration, posing a challenge for delivering genes or gene editors to these tissues in retinal gene therapy. In addition, LNPs have been traditionally composed of cationic lipids, which can lead to inflammatory and cytotoxic effects. Noncationic LNPs have been developed that offer minimal inflammatory and cytotoxic adverse effects [57]. In addition to LNPs, other lipid nanocarriers have also been explored [58].

#### 2.6.3. Inorganic Nanocarriers

Inorganic nanocarriers are usually made of inorganic materials such as gold, iron oxide, hydroxyapatite, and graphene. The utility of inorganic nanocarriers in targeted ocular delivery of RNA has been demonstrated. Examples of inorganic nanocarriers for RNA are antibody-conjugated polyethyleneimine-capped gold nanoparticles [59] and stimulus-responsive silica nanoparticles [60,61]. In addition to the potential of providing unique electrical and magnetic properties, the nature of inorganic nanocarriers allows precise designs with consistent size and properties. Although toxicity and solubility can be a challenge for these nanoparticles due to possible accumulation in the body, surface modifications can be used to reduce these issues.

#### 2.6.4. Biological Nanocarriers

An example of biological nanocarriers is exosomes. Exosomes are small vesicles enclosed by lipid bilayers that can cross biological membranes to deliver complex molecular cargoes. These extracellular vesicles are formed when multivesicular bodies within the cell fuse with the plasma membrane, releasing them into the extracellular matrix (ECM). By engineering exosomes to carry targeted therapeutic agents, their potential applications are beyond those of traditional nonbiological delivery systems. Exosomes also possess regenerative and anti-inflammatory capabilities and low immunogenicity. Recent developments include mesenchymal stem cell exosomes [62,63] and exosomes for gene delivery to treat various ocular diseases [64,65].

Another type of biological nanocarrier is adeno-associated viruses (AAV), which are the preferred vector for ocular gene therapies. The advantages of AAV include favorable safety profiles and efficient transduction capabilities. They have low immunogenicity and cytotoxicity relative to lentiviruses and adenoviruses. An example of AAV use is the treatment of RP associated with MERTK and choroideremia (CHM) [66,67].

#### 2.6.5. Others: RNA Nanoparticles

A recent development was the discovery of RNA-based nanoparticles using the packaging RNA three-way junction motif in the construction [68,69]. These RNA nanoparticles can be designed to have different sizes and shapes and have demonstrated potential in ocular delivery of RNA therapeutics [46,70,71].

## 3. RNA-Based Approach to Study the Mechanisms of Eye Diseases

### 3.1. Small Interfering RNA (siRNA)

siRNAs are short double-stranded RNA sequences that induce the degradation of target mRNA, thereby reducing the expression of specific genes [72]. siRNA can silence gene expression by directing sequence-specific cleavage of the target mRNA and interfering with gene transcription in the cells after transfection. siRNA and similar approaches have been used in research studies to investigate the factors that affect eye diseases and their mechanisms. Table 3 summarizes the siRNA to be highlighted in this section.

The first example is siRNA that targets apoptosis-stimulating proteins of p53 (ASPP2). ASPP2 can participate in epithelial–mesenchymal transition (EMT) of RPE. EMT of RPE is crucial in PVR. In the study, ASPP2-siRNA was used to transfect ARPE-19 cells to evaluate the role of ASPP2 in PVR and measure cell proliferation and migration ability [73]. PVR rat models were induced by ASPP2-siRNA-transfected ARPE-19 cells with intravitreal injection. Knocking down ASPP2 in ARPE-19 cells increased their proliferation, migration, and expression of mesenchymal markers and inflammatory cytokines. In the rat models, ASPP2 knockdown by ASPP2-siRNA treatment worsened PVR severity. These findings suggest that ASPP2 knockdown promotes EMT and exacerbates PVR progression through inflammatory and fibrosis cytokines.

β1-Integrins are crucial for angiogenesis, but their regulation in endothelial cells (EC) is not well understood. Brag2, a guanine nucleotide exchange factor, influences EC angiogenesis and was investigated [74]. In the study on integrins and angiogenesis, siRNA-mediated Brag2-silencing was found to reduce EC angiogenesis. Brag2-siRNA transfection differentially affected α5β1- and αVβ3-integrin function. Brag2 affected integrin recycling and endocytosis, promoting focal adhesion disassembly. Therefore, it was suggested that Brag2 silencing could decrease ischemia-induced RN and CNV.

siRNA was also used in the knockdown of SERPINA3K to investigate the role of SERPINA3K in DR [75]. Using an oxygen-induced retinopathy model, SERPINA3K was found to reduce retinal vascular leakage, leukostasis, and inflammation. SERPINA3K prevented hypoxia-induced decreases in occludin and blocked proinflammatory factors like VEGF, TNF-alpha, and ICAM-1. SERPINA3K also decreased ROS generation and increased antioxidant activity. The knockdown of SERPINA3K by siRNA led to an increase in VEGF and TNF-alpha. It was concluded that SERPINA3K was an anti-inflammatory factor, and reduced levels of SERPINA3K could contribute to retinal inflammation in DR.

In another example, siRNA targeting aurora kinase B (AURKB) was used to inhibit AURKB expression and transfect vascular endothelial cells [76]. Although AURKB is known to play a key role in chromosome segregation and tumor progression, the role of AURKB in pathological retinal angiogenesis remains elusive. Barasertib, an AURKB inhibitor, was used to compare with the anti-angiogenic effects of AURKB siRNA in vitro and in an oxygen-induced retinopathy (OIR) mouse model in vivo. Both AURKB siRNA and barasertib significantly inhibited endothelial cell proliferation, migration, and tube formation in vitro and reduced retinal angiogenesis in vivo, suggesting the role of AURKB in retinal angiogenesis. The results also suggested that AURKB is a promising therapeutic target for ocular neovascular diseases.

siRNA was also used to suppress canonical transient receptor potential (TRPC) channels in VEGF signaling [77]. VEGF is crucial for angiogenesis and contributes to retinopathy of prematurity. TRPC channels are important in VEGF signaling and their expression was found in the mouse retina. Among the TRPC channels, TRPC4 was chosen due to its upregulation in the hypoxic retina. It was found that TRPC4 suppression with siRNA transfection inhibited VEGF-induced migration and tube formation in human retinal cells. Intravitreal injection of siRNA against mTRPC4 reduced RN in a mouse model in vivo. These results suggested the role of TRPC4 in RN and TRPC4 suppression as a potential therapy for VEGF-induced RN.

Similarly, siRNA was used to target the VEGF gene and regulate the VEGF to pigment epithelium-deprived factor (PEDF) ratio (VEGF/PEDF) in attenuating OIR [78]. Retinal neovascularization involves multiple growth factors and the roles and interplay of VEGF (angiogenic) and PEDF (antiangiogenic) remain unclear. In the study, VEGF mRNA expression and VEGF levels were evaluated and found to be reduced by the VEGF-siRNA (psi-HI^TM^/EGFP/VEGF siRNA) in cell culture in vitro. In addition, siRNA treatment in the OIR mouse model in vivo decreased VEGF and the VEGF/PEDF ratio, significantly reducing neovascular tufts, avascular regions, and abnormal blood vessels. It was shown that VEGF-siRNA could effectively downregulate VEGF expression and the balance of VEGF/PEDF was important in regulating retinal angiogenesis. The VEGF-siRNA can attenuate RN by restoring the balance of VEGF/PEDF as a potential therapeutic target for RN.

In a study to explore the role of delta-like ligand 4 (Dll4) in CNV angiogenesis, siRNA was used to silence Dll4 expression [79]. This was based on evidence indicating that Dll4 participates in the HIF-1α–VEGF pathway involved in CNV angiogenesis. Using hypoxic RF/6A cells and a rat CNV model, Dll4 was found to enhance cell proliferation and tube formation but inhibit migration and invasion in hypoxic conditions. siRNA-mediated Dll4 silencing reduced these effects. The importance of Dll4 in CNV angiogenesis was demonstrated, suggesting that Dll4 could be a therapeutic target for CNV-related diseases.

In another study, siRNA was used to suppress intercellular adhesion molecule (ICAM)-1 expression in the murine retina [80]. ICAM-1 plays an important role in leukocyte migration. In the study, ICAM-1-specific plasmid siRNA was transfected into retinal cells using hydrodynamics-based transfection and intravitreal injection in vivo. It was found that ICAM-1 expression was suppressed in siRNA-treated cells compared to the controls. The results from downregulation of ICAM-1 by ICAM-1-specific siRNA suggested that the control of ICAM-1 expression (leukocyte infiltration) could be a potential target for retinal neovascular disease therapies.

In yet another study, lentiviral vector-mediated FoxO1 siRNA was used to examine the role of FoxO1 in interleukin-1β (IL-1β)-induced autostimulation in DR [81]. In the in vitro study, high glucose and IL-1β increased FoxO1 and IL-1β expression in human retinal cells. The high glucose level triggered IL-1β synthesis, which increased FoxO1 expression, leading to IL-1β autostimulation. In diabetic rats, FoxO1 and IL-1β levels were decreased by intravitreal injection of lentiviral vector-mediated FoxO1 siRNA. These results highlighted the functions of FoxO1 and IL-1β in DR and demonstrated that reducing FoxO1 could decrease inflammation in IL-1β-induced autostimulation.

Caveolin-1 expression is associated with endothelial membrane permeability but its role in RN is not completely understood. siRNA against caveolin-1 was therefore evaluated in a study of the blood-retina barrier and RN [82]. In this study, mice were exposed to high oxygen levels to induce retinopathy. Caveolin-1 expression was found to increase during hypoxia, correlating with albumin leakage. siRNA against caveolin-1 was found to reduce caveolin-1 mRNA and protein levels as well as neovascularization and albumin leakage in the mouse model. The results indicated that caveolin-1 was linked to endothelial barrier permeability and angiogenesis and played a significant role in RN. These findings suggested that caveolin-1 inhibition could be a promising treatment for ischemia-induced retinal diseases.

### 3.2. MicroRNA (miRNA) and Short Hairpin RNA (shRNA)

miRNA is a small non-coding RNA that normally binds to the 3′UTR region of mRNA by sequence complementarity [83]. miRNA can suppress the translation level of a protein encoded by mRNA in gene regulation. It can modulate cellular processes through transcriptional regulation. shRNA has a tight hairpin turn that can silence target gene expression by RNA interference [84]. By suppressing the translation of the target RNA, miRNA can be used to investigate the mechanisms of diseases and their development. Such understanding has allowed the development of miRNA therapeutics. Similarly, by silencing target gene expression, shRNA can be used to investigate the mechanisms of diseases. Table 4 lists the miRNA and shRNA to be discussed in this section.

Among the miRNAs investigated, miRNA miR-539-5p was identified as a regulator of CXCR7, a CXC chemokine receptor (CXCR) for stromal cell-derived factor-1 (SDF-1), and the effect of targeting miR-539-5p/CXCR7 in CNV-related diseases was evaluated [85]. It was found that the use of miR-539-5p mimic could block CXCR7 and reduce SDF-1-induced cell survival and tube formation in human retinal cells in vitro. miR-539-5p mimic could also decrease CNV leakage and lesion size in a rat model in vivo. The results suggested that the manipulation of miR-539-5p/CXCR7 levels could be important in therapies of CNV-associated diseases. In addition, a polymeric nanoparticle of PLGA grafted with internalizing arginine–glycine–aspartic acid (iRGD) peptide was developed for miR-539-5p (miR-539-iRGD-PLGA) [52]. The iRGD on the nanoparticle was used to target the alpha-v-beta-3 (αvβ3) integrin receptor, commonly overexpressed in the blood vessels of CNV. In cell culture, a larger reduction in cell viability with the iRGD-functionalized nanoparticle was observed, suggesting the potential of the polymeric nanoparticle.

In another study of miRNA and CNV, miR-188-5p expression was found to decrease in hypoxic mesenchymal stem cells (MSCs) and bone marrow-derived cells (BMCs) in CNV [86]. miR-188-5p targets matrix metalloproteinase 2 (MMP-2) and MMP-13, which were upregulated in CNV lesions. Transfecting cells with miR-188-5p mimic reduced MMP-2 and MMP-13 levels and tube formation. Intravitreal injection of miR-188-5p agomir reduced CNV severity and BMC migration in mice. These findings suggested that miR-188-5p regulated BMC contribution to CNV by targeting MMP-2 and MMP-13, and miR-188-5p could be a therapeutic target for CNV-related diseases.

Sirtuin 1 (SIRT1) is a protein involved in cell survival and metabolism in the cells forming the ocular structures, including the retina. In a study of SIRT1, miR-195 was evaluated for its role in regulating SIRT1 in DR [87]. The study showed that high glucose levels increased miR-195 and decreased SIRT1 in human retinal cells in vitro. Blocking miR-195 or overexpressing SIRT1 reversed these effects. In diabetic rats, miR-195 was upregulated, and blocking it improved SIRT1 levels. It was therefore suggested that miR-195 could regulate SIRT1, which contributed to retinal damage in DR.

SIRT1 was also the target mRNA of miR-23b-3p in the study of metabolic memory in DR [88]. The study illustrated that miR-23b-3p could affect the metabolic memory in DR with human endothelial retinal cells in vitro and retinal tissues from diabetic rats in vivo. In diabetic rats, it was found that high glucose levels increased miR-23b-3p expression. Reducing miR-23b-3p expression restored SIRT1 levels and alleviated high glucose metabolic memory effects. High glucose also promoted NF-κB p65 recruitment, enhancing miR-23b-27b-24-1 gene transcription. The importance of miR-23b-3p to regulate high-glucose-induced metabolic memory through an SIRT1-dependent pathway was highlighted in the study.

Using miR, a glucose-induced mechanism of miR-146a regulating extracellular matrix protein production was also revealed in a study related to DR [89]. The role of miR-146a in regulating extracellular matrix protein fibronectin (FN) production in diabetes was investigated in endothelial cells from retinal microvessels. High glucose levels decreased miR-146a and increased FN in endothelial cells. It was found that miR-146a mimic transfection could prevent these changes, while miR-146a antagomir transfection increased FN. The study further showed that intravitreal injection of miR-146a mimic restored miR-146a and decreased FN in diabetic rats.

In a study of ROP, miR-145-5p was used to investigate the role of long non-coding RNA taurine upregulated gene 1 (TUG1) in the disease using a mouse OIR model [90]. TUG1, miR-145-5p, and cellular communication network factor 1 (CCN1) were differentially expressed in retinal tissues of OIR mice. Knocking down TUG1 or overexpressing miR-145-5p reduced apoptosis, migration, and angiogenesis, and hence hypoxia-induced damage in retinal cells. miR-145-5p mimics reduced the degree of retinopathy. The study concluded that TUG1 regulated CCN1 expression by acting as a “molecular sponge” for miR-145-5p. Therefore, targeting TUG1/miR-145-5p/CCN1 could be a potential therapeutic approach for ROP.

The signal transducer and activator of transcription 3 (STAT3) can be an important transcription factor related to RN. In a study of miR-21 regulation, miR-21 was identified as a downstream effector of STAT3 activity in ischemic retina [91]. In hypoxic human retinal endothelial cells and a mouse OIR model, tissue inhibitor of matrix metalloproteinases 3 (TIMP3) expression was found to decrease alongside STAT3 activation and miR-21 upregulation. These findings suggested that miR-21 mediated STAT3 pro-angiogenic effects and could be a therapeutic target for preventing RN. In addition, miR-21 is involved in the inflammatory processes and is upregulated in DR and macular edema [94]. It was therefore concluded that miR-21 suppression could reduce angiogenesis and vascular leakage and prevent oxidative stress damage in DR.

For shRNA, a previous study used aquaporin 4 (AQP4) shRNA in the form of AQP4 shRNA^®^ lentiviral particles to investigate the role of AQP4 in DR in rats [92]. DR involves the altered expression of AQP4 in the retina. AQP4 knockdown worsened retinopathy, increasing retinal permeability, thickness, and inflammation. High glucose levels enhanced AQP4 expression, while AQP4 knockdown increased proinflammatory cytokines and VEGF. IL-1β and interleukin-6 (IL-6) suppressed AQP4 expression. The study suggested that AQP4 upregulation in diabetes is compensatory, and its downregulation exacerbates retinopathy. Therefore, regulating AQP4 could be a potential therapeutic strategy for DR.

shRNA was also used to investigate the role of Ras-related C3 botulinum toxin substrate 1 (Rac1) in hypoxia-induced RN in a mouse model [93]. In the study, vector-based shRNA targeting Rac1 (Rac1-shRNA) was employed. Intravitreal injection of pSUPER-Rac1-shRNA (constructed by the expression vector pSUPER RNAi system) reduced Rac1 expression and significantly inhibited RN. The study also found decreased levels of nuclear factor kappa B (NF-κB), hypoxia-inducible factor-1 alpha (HIF-1α), and VEGF in the retinal tissues, indicating that Rac1 regulated redox signaling in RN. From these results, Rac1 was suggested to be a potential therapeutic target for hypoxia-induced retinal neovascular diseases.

## 4. RNA-Based Approach to Treat Eye Diseases

### 4.1. Small Interfering RNA (siRNA) Treatments

siRNA can be used to treat posterior eye diseases by specifically targeting and silencing the genes related to the diseases after gene transcription. Table 5 lists the siRNAs that have been investigated for posterior eye disease treatments and presented in this review.

The first example is Bevasiranib (Cand5) for wet AMD [95]. Bevasiranib is designed to silence the gene that encodes VEGF, a protein responsible for promoting the growth of abnormal blood vessels in the retina in wet AMD. By inhibiting VEGF expression, the therapy aims to reduce neovascularization and prevent vision loss. Bevasiranib has shown promise in early-phase clinical trials (see Section 5), but the development was halted after a Phase 3 trial due to the lack of sufficient efficacy compared to existing anti-VEGF treatments like ranibizumab (Lucentis) and aflibercept (Eylea).

Another example of therapeutic siRNA for posterior eye disease is AGN211745 (Sirna-027), which targets VEGF receptor 1 (VEGFR-1) [96]. By silencing this receptor, the therapy aims to block the VEGF pathway and reduce the abnormal blood vessel growth associated with wet AMD. AGN211745 was one of the first siRNAs tested in clinical trials for wet AMD (see Section 5). Although early results showed good tolerability, efficacy was insufficient compared to current anti-VEGF therapies, and its development was discontinued [109,110].

PF-04523655 is a siRNA designed to target the RTP801 gene (a stress-response gene) and downregulate RTP801. RTP801 is also known as REDD1 (regulated in development and DNA damage responses 1), a stress-regulated protein involved in inflammation and cellular stress responses that contribute to retinal damage in DME and AMD [98]. Reducing the expression of RTP801 is intended to decrease inflammation, vascular leakage, and retinal cell death. PF-04523655 was tested in clinical trials for DME and AMD [111] (see Section 5).

Yet another example of siRNA for AMD is ISTH0036. ISTH0036 targets transforming growth factor-beta2 (TGF-β2) mRNA and exhibits potent activity in murine models of CNV to reduce neovascularization, vascular leakage, and fibrosis [100,112]. The safety of ISTH0036 was evaluated in glaucoma subjects undergoing trabeculectomy in a Phase 1 clinical trial. The clinical study investigated ISTH0036 to prevent excessive scarring and trabecular meshwork transformation in trabeculectomy [113]. As ISTH0036 has shown the potential for significant therapeutic benefits with pronounced effects in non-human primates, the human safety results supported Phase 2 development of ISTH0036 for treating wet AMD and DME [114] (see Section 5).

To improve intravitreal siRNA delivery, a nanocarrier of chitosan-hyaluronic acid nano-polyplexes was developed for VEGFR-2 siRNA [51]. The effects of hyaluronic acid composition on the fabrication of the nano-polyplexes and their properties were evaluated. VEGFR-2 siRNA was shown to down-regulate the VEGEF-2 expression in cells in vitro. In the animal model, the VEGFR-2 siRNA nano-polyplexes penetrated the retinal barriers and reduced the size of laser-induced CNV after intravitreal injection. These results suggested the nanocarrier as a viable method for intravitreal siRNA delivery in anti-VEGF therapies.

For RN, a vector-based VEGF isoform 165 (VEGF165)-targeted siRNA expression system (pSilencer^siVEGF^) was developed [101]. The siRNA was evaluated for its ability to inhibit VEGF165 expression in vitro and suppress RN in mice with ischemic retinopathy in vivo. Cells transfected with pSilencer^siVEGF^ were found to have reduced expression of the VEGF isoform. The reduction in RN in the animal model following intravitreal injection of pSilencer^siVEGF^ suggested its potential for treating ischemia-induced retinal diseases.

In addition to the common neovascular diseases, siRNA treatments have also been investigated for PVR, uveitis, neurodegeneration, NAION, and ocular hypertension and glaucoma. For PVR, siRNA protein kinase C-alpha (siRNA-PKCα) was evaluated for the effect on a mouse model [102]. Intravitreal injection of siRNA-PKCα was found to partly inhibit the cells in the process of PVR in the animal model. The result suggested that gene therapy with siRNA-PKCα silencing could provide a therapeutic target in the treatment of PVR.

For uveitis, a recombinant plasmid for inducible co-stimulator (ICOS) siRNA was constructed and shown to effectively downregulate the expression of ICOS for ocular inflammation [103]. In this study, intravitreal injection of the siRNA plasmid targeting ICOS was shown to suppress experimental autoimmune uveoretinitis (EAU) in rats.

For retinal ganglion cell (RGC) neuroprotective and axon regenerative effects, siRNA targeting the mTOR negative regulator RTP801 was investigated [104]. Intravitreal injection of siRTP801 was shown to provide RGC survival and neuroprotection in the optic nerve crush (ONC) animal model in vivo and retinal culture in vitro. These results suggested the potential of RTP801 knockdown by siRNA to promote RGC survival and axon elongation.

QPI-1007 is a siRNA that targets caspase 2 (CASP2), a gene involved in the apoptosis (programmed cell death) of RGC [105]. By inhibiting CASP2, QPI-1007 is designed to protect retinal ganglion cells from death in conditions such as NAION, which leads to vision loss due to optic nerve ischemia. QPI-1007 was suggested as a treatment for NAION and was evaluated in clinical trials [106,115] (see Section 5).

For ocular hypertension and glaucoma, SYL040012 is a siRNA that targets the β2-adrenergic receptor (ADRB2) gene, which is involved in regulating IOP. By inhibiting this gene, SYL040012 can lower IOP, a key factor in the progression of glaucoma and related optic nerve damage. SYL040012 was evaluated for its effect on IOP and tolerability in clinical trials [107,116] (see Section 5).

Despite not being a posterior eye disease, it is worth mentioning a siRNA drug in Phase 3 clinical trials. Tivanisiran (SYL1001) is a siRNA targeting the transient receptor potential cation channel subfamily V member 1 (TRPV1), which is involved in mediating ocular surface pain and inflammation in dry eye disease [108]. By silencing the TRPV1 receptor, SYL1001 was evaluated in clinical trials to reduce discomfort and inflammation associated with dry eye disease [117,118].

### 4.2. RNA Interference by microRNA (miRNA) and Short Hairpin RNA (shRNA)

In addition to siRNA, miRNA and shRNA can be used as therapeutic agents in the treatment of posterior eye diseases. By controlling gene expression, RNA interference by miRNA and shRNA can be a potential therapeutic. Table 6 summarizes the miRNA and shRNA to be discussed in this section.

An example of miRNA as a potential target of therapeutics is miR200-b. miR200-b was examined for its potential as an anti-angiogenic factor on VEGF receptor 2 (VEGF-2) expression for DR treatment [119]. An inverse correlation between the level of expression of miR200-b and VEGFR-2 was observed. The delivery of miR200-b reduced protein levels of VEGFR-2 and suppressed angiogenesis in a mouse model.

For shRNA, lentiviral vectors (Lv) Lv-shRNA-EphB4 were used to transfect and knockdown erythropoietin-producing hepatocellular carcinoma receptors B4 (EphB4) [120]. The knockdown of EphB4 expression inhibited CNV in an animal model. In another example, shRNA that inhibited Ras-related C3 botulinum toxin substrate 1 (Rac1-shRNA) was evaluated for its effect on RN in a rat model [121]. The shRNA was transfected into HeLa cells and in rats. It was found that the shRNA reduced the area of neovascularization in the animal model, suggesting that silencing Rac1 expression can inhibit RN.

In addition to single-target intervention, shRNA was also evaluated in dual-target treatment. A transfection reagent-treated non-viral vector carrying anti-connective tissue growth factor (CTGF) shRNA was used in conjunction with anti-VEGF treatment [122]. It was hypothesized that the anti-VEGF would elevate CTGF mRNA and cause up-regulation of CTGF in the retina, increasing the risk of fibrosis. A dual-target treatment of CTGF shRNA and anti-VEGF ranibizumab was therefore proposed and evaluated for DR.

### 4.3. RNA Aptamers

Aptamers are short, single-stranded artificial nucleic acids (DNA or RNA). These oligonucleotides bind to specific targets with high affinity, similar to proteins. For example, aptamers can bind to proteins by recognizing their tertiary or quaternary structures [123]. Aptamers can also bind to small molecules [124]. They have been explored as therapeutic agents for various diseases, including eye conditions. These nucleic acid-based molecules offer advantages such as low immunogenicity and the potential for chemical modification to increase their stability. The RNA aptamers to be reviewed are presented in Table 7.

Pegaptanib (Macugen) is an aptamer developed for wet AMD [125]. The RNA aptamer is modified by PEGylation to improve its stability and vitreous half-life. It specifically binds to and inhibits the VEGF isoform 165 (VEGF165), a key molecule in the process of angiogenesis that leads to the growth of abnormal blood vessels in the retina. This reduces the neovascularization associated with wet AMD and prevents vision loss. Pegaptanib was one of the first aptamers approved for clinical use and the first aptamer approved for wet AMD (FDA approval in 2004). However, newer anti-VEGF therapies (e.g., ranibizumab and aflibercept) have largely replaced pegaptanib in clinical practice due to their higher efficacy.

A recent development was the approval of avacincaptad pegol (brand name Izervay and earlier as Zimura or ARC1905 in clinical trials [134]) by the FDA for the treatment of GA in dry AMD. Avacincaptad pegol is a PEGylated oligonucleotide (anti-C5 aptamer) that targets complement component 5 (C5) protein, which plays a role in the inflammatory processes associated with GA [126]. By inhibiting C5, the aptamer is designed to reduce complement-mediated inflammation and tissue damage in the retina. Intravitreal injection of the C5 inhibitor was observed to reduce GA lesion growth in dry AMD treatment [135].

RBM-007 is an RNA aptamer that targets fibroblast growth factor 2 (FGF2), a growth factor implicated in both angiogenesis and fibrosis in wet AMD [127]. By inhibiting FGF2, RBM-007 aims to reduce both the formation of abnormal blood vessels and the fibrotic scarring that can result from wet AMD. The safety and efficacy of RBM-007 were evaluated in clinical trials [136,137] (see Section 5).

Another antiangiogenic aptamer is AS1411 [128,129]. The nucleolin-binding aptamer was found to reduce proliferation, cell migration, and tube formation in human umbilical vein endothelial cells (HUVEC) in vitro, inhibit branch formation in the rat aortic ex vivo, and inhibit RN in OIR and laser-induced CNV animal models in vivo. In addition, topical application of AS1411 was shown to be effective in the CNV animal model. It was suggested that nucleolin was a target for wet AMD and that AS1411 could be developed for anti-angiogenic treatment, e.g., wet AMD.

Although not an RNA aptamer, it is noteworthy to mention E10030 (Fovista) in dual antagonism of platelet-derived growth factor (PDGF) and VEGF for AMD (see Section 4.5). E10030 is a DNA aptamer that targets PDGF-B, a growth factor involved in the recruitment of pericytes that stabilize abnormal blood vessels in wet AMD [138]. By inhibiting PDGF-B, Fovista is designed to strip pericytes from neovascular vessels, making them more susceptible to anti-VEGF treatments. Fovista was evaluated in Phase 3 clinical trials [139].

### 4.4. Antisense Oligonucleotides (ASO)

Antisense oligonucleotides (ASO) are short, synthetic strands of nucleotides designed to bind to specific mRNA sequences and modulate gene expression [131]. This approach has been successfully applied to treat ocular conditions. ASO can also interfere with protein translation by mRNA degradation [140]. The first FDA-approved ASO for posterior eye disease is fomivirsen (Vitravene). Fomivirsen is an antiviral drug for CMV. In addition, there are experimental ASOs under investigation for eye diseases, especially those with a genetic basis. Table 7 lists the ASO to be discussed in this section.

QR-110 (Sepofarsen) is an example of an ASO that has been under clinical trials [130]. It shows promising results in improving visual function in patients with LCA10 [141]. LCA10 is caused by a mutation in the CEP290 gene, which affects the normal splicing of pre-mRNA, resulting in a defective protein. Sepofarsen is an ASO that binds to the mutated region of the gene and promotes correct splicing, enabling the production of a functional CEP290 protein. QR-110 has been evaluated for its efficacy, safety, and systemic exposure after intravitreal administration in subjects with LCA in clinical trials [142] (see Section 5).

Another example is allele-specific ASO-mediated knockdown of mutant P23H rhodopsin expression for autosomal dominant RP (adRP) [143]. IONIS-RHO-2.5Rx (QR-1123) is an ASO targeting the rhodopsin (RHO) gene (i.e., rhodopsin mutation). Rhodopsin mutations lead to adRP, where defective rhodopsin proteins cause retinal degeneration. IONIS-RHO-2.5Rx is designed to reduce the expression of the mutant RHO gene, thereby slowing the progression of the disease. IONIS-RHO-2.5Rx is in early-phase clinical trials, showing the potential in reducing retinal degeneration and improving patient outcomes [144].

Yet another example is QR-421a, an ASO in clinical trials for Usher syndrome type 2A [132]. Usher syndrome causes both hearing loss and vision loss due to the mutations in the USH2A gene (particularly, exon 13 mutations) [145]. QR-421a targets the mutated exon, skipping the faulty region and allowing the production of a functional protein. Preliminary data of QR-421a have shown promising effects on preserving vision in patients with these mutations [132]. QR-421a was evaluated in clinical trials for its efficacy, safety, and tolerability for RP [146,147] (see Section 5).

To improve ASO delivery, the effectiveness of a cationic nanoemulsion was assessed using animal models of corneal neovascularization and ROP with ODN17, an ASO targeting VEGF-R2 [58]. The results of corneal neovascularization will not be discussed here. For ROP, ODN17 was found to reduce RN, and the nanoemulsion significantly improved the inhibition effect of ODN17. The results demonstrated the effect of cationic nanoemulsion for enhancing the delivery of ASO in ocular therapies.

For PVR treatment, c-fos antisense oligonucleotide (c-fos-AS-ON) blocked the expression of c-fos and inhibited cultured human RPE cell proliferation [133]. It was found that the injection of c-fos-AS-ON reduced the prevalence of experimental PVR.

### 4.5. Other Therapeutics

Although not the focus of the present review, this section provides a few examples of other therapeutics such as DNA aptamers and AAV gene therapies for the treatment of posterior eye diseases. Table 8 shows an example of a DNA aptamer and three examples of AAV gene therapies, which have been evaluated for wet AMD and choroideremia (CHM) treatments. The gene therapies involve AAV as vectors to deliver therapeutic genes into target cells and insert the genetic material at specific sites in the genome. Fovista (E10030) is a DNA aptamer that binds to PDGF-B in combination therapy with Lucentis for wet AMD [138,139] (see Section 4.3). For AAV gene therapies, ixoberogene soroparvovec (ixo-vec) [148] and RGX-314 (Regenxbio) [149] target the genes to encode anti-VEGF proteins and NSR-REP1 [150] to the Rab escort protein 1 (REP1) in the treatment of wet AMD.

### 4.6. Challenges and Future Direction in RNA Development for Eye Diseases

Despite the promising potential of RNA therapeutics, several challenges remain. These challenges include: (a) low efficiency delivery, (b) short duration of effect, and (c) safety and off-target effects. The delivery of RNA to the retina is difficult due to the eye’s anatomical barriers. As a result, the delivery of RNA therapeutics to specific tissues in the eye, particularly the retina, remains a significant challenge. As discussed in Section 2.3 and Section 2.4, current delivery methods include intravitreal and subretinal injections, but better options are preferred. More research is needed to develop less invasive and more effective methods for RNA delivery. RNA therapeutics can have a short duration of action and require repeated dosing as their effects are often temporary when they degrade over time. For example, the transient nature of ASO and siRNA therapy may require frequent administration. Although efforts are underway to improve the duration of the effects of these RNA therapeutics, this remains a challenge. Furthermore, ensuring the specificity of RNA therapeutics to avoid unintended gene silencing or adverse effects on other cellular processes is crucial and can be difficult. Particularly, safety can be a concern with off-target effects and immune responses with siRNA therapies. Advancements in molecular design and delivery systems have the potential to address these issues.

## 5. Clinical Trials of RNA Therapeutics for Eye Disease Treatments

Drugs based on the principle of RNA interference hold great promise in the treatment of posterior eye diseases, and some of them have reached the clinical stage. This section provides a summary of these RNA therapeutics. Three RNA-based therapeutics for the posterior eye have been approved by the FDA: fomivirsen, pegaptanib, and avacincaptad pegol. Fomivirsen is an ASO and pegaptanib and avacincaptad pegol are RNA aptamers (also see Section 4.3 and Section 4.4). Fomivirsen (Vitravene) is an ASO for CMV in immunocompromised patients, and the brand name Vitravene is discontinued. Pegaptanib (Macugen) is an inhibitor of VEGF for wet AMD. Avacincaptad pegol (Izervay, formerly Zimura) is an inhibitor of complement C5 for GA. The other RNA therapeutics have only reached the clinical trial stages. Table 9 lists the RNA-based therapeutics for posterior eye diseases, CNV, AMD, DME, GA, RP, and glaucoma. Drugs for CNV, AMD, and DME include Cand5 [151,152], AGN211745 [97,153], PF-04523655 [98,99,154], RBM-007 [136], SYL1801 [155], HG202 [156,157] and ISTH0036 [113]. As siRNA, Cand5 silences the mRNA encoding of VEGFA for DME and wet AMD and PF-04523655 targets the RTP801 gene for DME. OLX10212 is a drug of cell-penetrating asymmetric siRNA (cp-asiRNA) targeting the inflammation pathways in the development of GA and AMD [158]. SYL1801 is an siRNA eye drop for wet AMD. As an ASO, Sepofarsen (QR-110) was evaluated for its efficacy and safety for LCA due to the mutation in the CEP290 gene [142]. QR-1123 was evaluated for its potential on adRP due to the P23H mutation in the RHO gene [144], and Ultevursen (QR-421a) for RP due to the mutations in exon 13 of the USH2A gene [132,146,147]. For glaucoma, SYL040012 was used to control elevated IOP [116]. For NAION, QPI-1007 was used to protect retinal ganglion cells from death [115].

## 6. Conclusions

The emergence of siRNA, shRNA, and miRNA has allowed the investigation of the mechanisms of posterior eye diseases by targeting nucleic acids, e.g., RNA interference. These diseases span neovascularization, genetic defects, scarring from retinal detachment, glaucoma, and inflammation in the posterior segment of the eye. A mechanistic approach, utilizing siRNA, shRNA, and miRNA, can provide the knowledge base and methods needed to improve posterior eye disease treatments. By utilizing RNA technology and understanding disease mechanisms in these basic and preclinical studies, interventions on these mechanisms using RNA-based therapeutics can be developed.

RNA therapeutics represent an innovative approach to treating genetic eye diseases by targeting underlying molecular causes. As clinical trials advance, these therapies have the potential to change the treatment landscape for patients with inherited retinal disorders and other degenerative eye conditions. siRNA, shRNA, miRNA, and ASO therapies offer a novel and targeted approach to treating a variety of eye diseases by directly silencing disease-driving genes. RNA aptamers bind to specific targets with high affinity and have the advantage of low immunogenicity compared to proteins. Although many RNA-based therapies are still in experimental or clinical trial stages, they hold great promise for conditions such as DR, DME, AMD, glaucoma, and inherited retinal disorders. Challenges such as effective delivery methods and long-term safety will need to be addressed before these treatments can become widely available. The continuing advancements in delivery systems, efficacy, and safety will be critical for bringing these treatments to clinical use.

## Figures and Tables

**Table 1:** Eye diseases related to the RNA studies reviewed in this paper.

Type of Diseases	Disease
Neovascularization-Related Diseases -- These diseases involve abnormal growth of blood vessels in the retina or choroid, often driven by VEGF. -- RN is commonly seen in DR, and CNV is normally associated with AMD.	Diabetic Retinopathy (DR) -- Non-Proliferative DR (NPDR) -- Proliferative DR (PDR) Diabetic Macular Edema (DME) Age-Related Macular Degeneration (AMD) -- Dry AMD -- Wet AMD Choroidal Neovascularization (CNV) Retinal Neovascularization (RN) Retinopathy of Prematurity (ROP)
Inflammatory Diseases -- These involve immune-mediated inflammation in the uveal tract.	Uveitis -- Anterior Uveitis -- Intermediate Uveitis -- Posterior Uveitis
Degenerative and Genetic Diseases -- These diseases involve progressive retinal degeneration, often due to inherited mutations.	Retinitis Pigmentosa (RP)
Ischemic and Optic Nerve Diseases -- These conditions result from restricted blood flow or damage to the optic nerve. The damage can be associated with elevated intraocular pressure (IOP).	Non-Arteritic Anterior Ischemic Optic Neuropathy (NAION) Glaucoma
Structural and Proliferative Disorders -- These conditions involve anatomical disruption and abnormal cellular proliferation.	Proliferative Vitreoretinopathy (PVR)

**Table 2:** Modes of drug delivery for the posterior segment of the eye [16,17,18,19].

Mode	Characteristic	Advantage	Disadvantage
Topical	-- Most common for the anterior segment	-- Safe -- Convenient	-- Not effective for the posterior segment -- Some systemic exposure
Systemic	-- Limited by the BRB -- Can be used when a systemic effect (not in the eye) is also needed	-- Convenient	-- Not very effective for the posterior segment -- Most systemic exposure
Periocular	-- Includes: subconjunctival, sub-Tenon’s, retrobulbar, peribulbar, posterior juxtascleral -- Transscleral route	-- Low injection risk -- Low systemic exposure	-- Limited effectiveness for the posterior segment -- Some systemic exposure -- Invasive -- Local adverse effects
Intravitreal	-- Most common for the posterior segment	-- Effective for the posterior segment -- Less invasive than subretinal and suprachoroidal	-- Moderate risk -- Invasive -- Ophthalmology specialist required -- Can lead to severe adverse events
Subretinal	-- Direct administration to the vicinity close to the retina	-- Most effective -- Local	-- High risk (relative to the other modes) -- Invasive -- Ophthalmology specialist and special skills required -- Can lead to severe adverse events
Suprachoroidal	-- Direct administration to the vicinity close to the choroid	-- Second most effective -- Less invasive than subretinal -- Local	-- High risk (relative to the other modes) -- Invasive -- Ophthalmology specialist and special skills required -- Can lead to severe adverse events

**Table 3:** siRNA in the studies on the mechanisms of eye diseases.

siRNA	Target; Disease	Delivery Method ^a^	Reference
ASPP2-siRNA	ARPE-19 cell transfection, ASPP2 knockdown that worsens PVR via inflammatory and fibrosis cytokines; PVR	In vitro: HiPerFect Transfection Reagent; In vivo: Intravitreal, transfected cells	[73]
Brag2-siRNA	Brag2-siRNA transfection, Brag2-silencing, angiogenesis in endothelial cells; RN and CNV	In vitro: Transfection reagent Lipofectamine RNAiMAX; In vivo: Intravitreal, in vivo-jetPEI™ reagent	[74]
siRNA SERPINA3K	Knockdown of SERPINA3K, overexpression of VEGF and TNF-alpha; DR	In vitro: Transfection reagent siPORT	[75]
AURKB siRNA	Inhibiting aurora kinase B (AURKB) expression, transfecting vascular endothelial cells, inhibiting endothelial cell proliferation; RN and CNV	In vitro: Transfection reagent Lipofectamine^®^ 2000	[76]
mTRPC4 siRNA	TRPC4 suppression, inhibiting VEGF-induced pathways; RN	In vitro: Transfection reagent Lipofectamine RNAiMAX; In vivo: Intravitreal	[77]
VEGF-siRNA (psi-HI^TM^/EGFP/VEGF siRNA)	Targeting VEGF gene, reducing VEGF mRNA and protein expression, and regulating VEGF to pigment epithelium-deprived factor (PEDF) ratio; RN	In vitro: Transfection reagent Lipofectamine™ 2000; In vivo: Intravitreal, Lipofectamine™ 2000	[78]
Dll4 siRNA	Dll4 silencing, opposite effects of enhancing Dll4 expression, and regulating angiogenesis; CNV	In vitro: Transfection reagent Lipofectamine™ 2000	[79]
ICAM-1-specific plasmid siRNA	Suppressing intercellular adhesion molecule (ICAM)-1 expression, inhibiting leukocyte infiltration; RN	In vivo: (1) Systemic injection/ Hydrodynamics-based transfection (HT); (2) Intravitreal	[80]
FoxO1 siRNA	Increasing expressions of FoxO1 and IL-1β; DR	In vitro: Transfection by lentiviral vector In vivo: Intravitreal, transfection by lentiviral vector	[81]
Caveolin-1 siRNA	Reducing caveolin-1 mRNA and protein; RN	In vivo: Intravitreal, Lipofectamine™ 2000	[82]

^a^ Cell culture in vitro; animal model in vivo.

**Table 4:** miRNA and shRNA in the studies on the mechanisms of eye diseases.

	Target; Disease	Delivery Method ^a^	Reference
MicroRNA:			
miR-539-5p	CXCR7 for stromal cell-derived factor-1 (SDF-1); CNV	In vitro: Transfection reagent Lipofectamine 2000 or iRGD-PLGA nanoparticle (miR-539-iRGD-PLGA); In vivo: Intravitreal	[52,85]
miR-188-5p	Regulating the contribution of bone marrow-derived cells (BMCs) to CNV; CNV	In vitro: Transfection reagent Lipofectamine 2000; In vivo: Intravitreal	[86]
miR-195	Regulating sirtuin 1 (SIRT1); DR	In vitro: Transfection reagent Lipofectamine; In vivo: Intravitreal	[87]
miR-23b-3p and SIRT1 siRNA	Transfection with miR-23b-3p inhibitor, SIRT1-dependent signaling pathway; DR	In vitro: Transfection reagent Lipofectamine; In vivo: Intravitreal	[88]
miR-146a	FN 3′-untranslated region (UTR); diabetes related ocular diseases	In vitro: Transfection reagent Lipofectamine; In vivo: Intravitreal	[89]
miR-145-5p	RNA taurine upregulated gene 1 (TUG1); ROP	In vitro: Transfection reagent Lipofectamine; In vivo: Intravitreal	[90]
miR-21	Downstream effector of signal transducer and activator of transcription 3 (STAT3) activity; RN	In vitro: Transfection reagent Lipofectamine; In vivo: Intraorbital injection	[91]
shRNA:			
AQP4 shRNA	Transfection in rMC-1 cell; inflammation induced by high glucose	In vitro: transfection by lentiviral vector; In vivo: Intravitreal	[92]
pSUPER-Rac1-shRNA	Retinal Rac1 gene expression; hypoxia-induced RN	In vitro: Transfection reagent Lipofectamine; In vivo: Intravitreal, Lipofectamine 2000	[93]

^a^ Cell culture in vitro; animal model in vivo.

**Table 5:** siRNA as therapeutics in the studies that evaluate their effects on eye diseases.

siRNA	Target; Disease	Delivery Method ^a^	Reference
Bevasiranib (Cand5)	VEGF; wet AMD	In vitro: Naked RNA; In vivo: Intravitreal	[95]
AGN211745 (Sirna-027)	VEGFR-1; wet AMD	In vitro: Transfection reagent Lipofectamine 2000; In vivo: Intravitreal/ periocular	[96,97]
PF-04523655	RTP801 (REDD1 gene); DME and AMD	In vitro: Naked RNA; In vivo: Intravitreal	[98,99]
ISTH0036	TGF-β mRNA; DME and AMD	In vitro: Naked LNA-modified 14-mer antisense; In vivo: Intravitreal In vivo: Intravitreal	[100]
VEGFR-2 siRNA	VEGFR-2; CNV	In vitro: Transfection reagent Lipofectamine; In vivo: Intravitreal, nano-polyplexes of chitosan and hyaluronic acid	[51]
pSilencer^siVEGF^	Inhibiting VEGF(165) expression; RN	In vitro: Transfection reagent Lipofectamine 2000; In vivo: Intravitreal, liposome–pSilencer siVEGF complex	[101]
siRNA-PKCα (protein kinase C-alpha)	Markers for glia cells, fibroblast cells, RPE cells, and Müller cells in PVR; PVR	In vitro: Transfection reagent Lipofectamine 2000; In vivo: Intravitreal	[102]
ICOS siRNA	ICOS gene; uveoretinitis	In vitro: Transfection reagent Lipofectamine 2000; In vivo: Intravitreal	[103]
siRTP801	mTOR negative regulator RTP801, enabling RGC survival; ONC neuroprotection	In vitro: Transfection reagent Lipofectamine 2000; In vivo: Intravitreal	[104]
QPI-1007	Caspase 2 (CASP2); NAION	In vitro: Naked RNA; In vivo: Intravitreal	[105,106]
SYL040012	β2-Adrenergic receptor gene; ocular hypertension	In vivo: Topical eye drops, naked siRNA targeting ADRB2	[107]
Tivanisiran (SYL1001)	TRPV1; dry eye disease	In vivo: Topical eye drops, naked siRNA duplex	[108]

^a^ Cell culture in vitro; animal model or human in vivo.

**Table 6:** miRNA and shRNA as therapeutics in the studies that evaluate their effects on eye diseases.

	Target; Disease	Delivery Method ^a^	Reference
MicroRNA:			
miR200-b	VEGF receptor 2 (VEGFR-2) expression; DR	In vitro: Transfection reagent Lipofectamine 2000; In vivo: Intravitreal	[119]
shRNA:			
lentiviral vectors (Lv) Lv-shRNA-EphB4	Transfection of Lv-shRNA-EphB4, knockdown of EphB4, and downregulating EphB4 mRNA; CNV	In vitro: Lentiviral vector (expressing shRNA targeting EphB4); In vivo: Intravitreal	[120]
Rac1-shRNA	Rac1 expression; RN	In vitro: Transfection reagent Lipofectamine 2000; In vivo: Intravitreal	[121]
CTGF shRNA	Connective tissue growth factor (CTGF); diabetic retina	In vitro: Transfection reagent TransIT-TKO^®^; In vivo: Intravitreal	[122]

^a^ Cell culture in vitro; animal model in vivo.

**Table 7:** RNA aptamers and ASO as therapeutics in the studies that evaluate their effects on eye diseases.

	Target; Disease	Delivery Method ^a^	Reference
Aptamer:			
Pegaptanib	VEGF165 isoform; wet AMD	In vivo: Intravitreal	[125]
Zimura, ARC1905	Complement component 5 (C5); AMD	In vivo: Intravitreal	[126]
RBM-007	Fibroblast growth factor 2 (FGF2); wet AMD	In vitro: Naked RNA; In vivo: Intravitreal	[127]
AS1411	Anti-angiogenic nucleolin-binding aptamer; RN and CNV	In vitro: Naked RNA; In vivo: (1) Topical eye drops; (2) Intravitreal	[128,129]
ASO:			
QR-110 (Sepofarsen)	CEP290 gene; LCA	In vivo: Intravitreal	[130]
IONIS-RHO-2.5Rx (QR-1123)	RHO gene; autosomal dominant RP	In vivo: Intravitreal, naked RNA and chemically modified ASO (gapmer design)	[131]
QR-421a (Ultevursen)	USH2A gene; Usher syndrome, RP	In vitro: naked RNA; In vivo: Microinjection into the eye	[132]
ODN17	VEGF-R2, ROP	In vivo: Intravitreal, cationic nanoemulsion	[58]
C-fos-AS-ON	C-Fos; PVR	In vitro: Transfection reagent Lipofectin; In vivo: Intravitreal	[133]

^a^ Cell culture in vitro; animal model or human in vivo.

**Table 8:** Other therapeutics in studies that evaluate their effects on eye diseases.

Type	RNA/DNA/Others	Target; Disease	Delivery Method ^a^	Reference
DNA Aptamer	E10030	PDGF-B; wet AMD	In vivo: Intravitreal, PEGylation	[138,139]
AAV gene therapy	Ixoberogene soroparvovec (ixo-vec)	Encodes for an anti-VEGF protein; wet AMD	In vivo: Intravitreal, AAV.7m8 (engineered adeno-associated virus)	[148]
AAV gene therapy	RGX-314 (Regenxbio)	Encodes for an anti-VEGF protein; wet AMD	In vivo: Subretinal, AAV8 (adeno-associated virus serotype 8)	[149]
AAV gene therapy	NSR-REP1	Replenishing REP1 (Rab Escort Protein 1); CHM	In vivo: Subretinal, AAV2.REP1 (adeno-associated virus serotype 2 encoding Rab escort protein 1)	[66,150]

^a^ Animal model or human in vivo.

**Table 9:** RNA therapeutics evaluated in clinical trials for posterior eye disease treatments ^a^.

Drug	Clinical Trials and Descriptions	Year Completed/Terminated	Reference ^b^
Bevasiranib (Cand5)	Phases 2 and 3, siRNA silencing the mRNA encoding of VEGFA for DME and wet AMD	2007	[151,152]
AGN211745 (siRNA-027)	Phase 2, targeting VEGFR1 for subfoveal CNV associated with AMD	2009	[97,153]
PF-04523655	Phase 2, targeting the RTP801 gene alone and in combination with ranibizumab, and ranibizumab alone in DME	2013	[98,99,154]
SYL040012	Phase 2, treating elevated IOP for glaucoma	2016	[116]
ISTH0036	Phase 1, targeting TGF-β2 in glaucoma undergoing trabeculectomy	2017	[113]
QPI-1007	Phase 2/3, protecting retinal ganglion cells from death in acute NAION	2019	[115]
RBM-007	Phase 2, RBM-007 alone and RBM-007 with Eylea with wet AMD (TOFU)	2021	[136]
SYL1801	Phase 1, eye drop for the prevention and/or control of wet AMD	2021	[155]
OLX10212	Phase 1, targeting inflammation pathways in the development of GA and AMD	2024	[158]
HG202	Phase 1, CRISPR/Cas13 RNA-editing, delivered through AAV vector, to partially knock down the expression of VEGFA for wet AMD	2025, 2031 (estimated)	[156,157]
Sepofarsen (QR-110)	Phase 2/3, intravitreal injection in patients of LCA due to mutation in the CEP290 gene, to evaluate the efficacy, safety, and systemic exposure of QR-110	2022	[142]
QR-1123	Phase 1/2, targeting the mutant P23H mRNA to reduce the expression of the P23H protein selectively for adRP due to the mutation in the RHO gene	2022	[144]
Ultevursen (QR-421a)	Phase 2/3, inducing exon skipping, for Usher syndrome and RP caused by mutations in the USH2A gene	2022, 2021	[132,146,147]

^a^ FDA-approved drugs fomivirsen (Vitravene), pegaptanib (Macugen), and avacincaptad pegol (Izervay) are not included in the table. ^b^ For multiple clinical trials, only one or two examples are listed.

## References

[1] Williams M.A., McKay G.J., Chakravarthy U. (2014). Complement inhibitors for age-related macular degeneration. Cochrane Database Syst. Rev..

[2] Ni X., Castanares M., Mukherjee A., Lupold S.E. (2011). Nucleic acid aptamers: Clinical applications and promising new horizons. Curr. Med. Chem..

[3] Gupta A., Kafetzis K.N., Tagalakis A.D., Yu-Wai-Man C. (2021). RNA therapeutics in ophthalmology—Translation to clinical trials. Exp. Eye Res..

[4] Halloy F., Biscans A., Bujold K.E., Debacker A., Hill A.C., Lacroix A., Luige O., Stromberg R., Sundstrom L., Vogel J. (2022). Innovative developments and emerging technologies in RNA therapeutics. RNA Biol..

[5] Booth B.J., Nourreddine S., Katrekar D., Savva Y., Bose D., Long T.J., Huss D.J., Mali P. (2023). RNA editing: Expanding the potential of RNA therapeutics. Mol. Ther..

[6] Congdon N., O’Colmain B., Klaver C.C., Klein R., Muñoz B., Friedman D.S., Kempen J., Taylor H.R., Mitchell P., Eye Diseases Prevalence Research Group (2004). Causes and prevalence of visual impairment among adults in the United States. Arch. Ophthalmol..

[7] Kanwar J.R., Shankaranarayanan J.S., Gurudevan S., Kanwar R.K. (2014). Aptamer-based therapeutics of the past, present and future: From the perspective of eye-related diseases. Drug Discov. Today.

[8] Witmer A.N., Vrensen G.F.J.M., Van Noorden C.J.F., Schlingemann R.O. (2003). Vascular endothelial growth factors and angiogenesis in eye disease. Prog. Retin. Eye Res..

[9] Apte R.S., Chen D.S., Ferrara N. (2019). VEGF in signaling and disease: Beyond discovery and development. Cell.

[10] Asahara T., Chen D., Takahashi T., Fujikawa K., Kearney M., Magner M., Yancopoulos G.D., Isner J.M. (1998). Tie2 receptor ligands, angiopoietin-1 and angiopoietin-2, modulate VEGF-induced postnatal neovascularization. Circ. Res..

[11] White R.R., Shan S., Rusconi C.P., Shetty G., Dewhirst M.W., Kontos C.D., Sullenger B.A. (2003). Inhibition of rat corneal angiogenesis by a nuclease-resistant RNA aptamer specific for angiopoietin-2. Proc. Natl. Acad. Sci. USA.

[12] Akwii R.G., Sajib M.S., Zahra F.T., Mikelis C.M. (2019). Role of Angiopoietin-2 in vascular physiology and pathophysiology. Cells.

[13] Oliner J., Min H., Leal J., Yu D., Rao S., You E., Tang X., Kim H., Meyer S., Han S.J. (2004). Suppression of angiogenesis and tumor growth by selective inhibition of angiopoietin-2. Cancer Cell.

[14] Yu X., Sha J., Xiang S., Qin S., Conrad P., Ghosh S.K., Weinberg A., Ye F. (2016). Suppression of KSHV-induced angiopoietin-2 inhibits angiogenesis, infiltration of inflammatory cells, and tumor growth. Cell Cycle.

[15] Shirley M. (2022). Faricimab: First approval. Drugs.

[16] Edelhauser H.F., Rowe-Rendleman C.L., Robinson M.R., Dawson D.G., Chader G.J., Grossniklaus H.E., Rittenhouse K.D., Wilson C.G., Weber D.A., Kuppermann B.D. (2010). Ophthalmic drug delivery systems for the treatment of retinal diseases: Basic research to clinical applications. Investig. Ophthalmol. Vis. Sci..

[17] Ghate D., Edelhauser H.F. (2006). Ocular drug delivery. Expert. Opin. Drug Deliv..

[18] Lee S.S., Robinson M.R. (2009). Novel drug delivery systems for retinal diseases: A review. Ophthalmic Res..

[19] Hartman R.R., Kompella U.B. (2018). Intravitreal, subretinal, and suprachoroidal injections: Evolution of microneedles for drug delivery. J. Ocul. Pharmacol. Ther..

[20] Araujo J., Gonzalez E., Egea M.A., Garcia M.L., Souto E.B. (2009). Nanomedicines for ocular NSAIDs: Safety on drug delivery. Nanomedicine.

[21] Zimmer A., Kreuter J. (1995). Microspheres and nanoparticles used in ocular delivery systems. Adv. Drug Deliv. Rev..

[22] Gaudana R., Jwala J., Boddu S.H., Mitra A.K. (2009). Recent perspectives in ocular drug delivery. Pharm. Res..

[23] Del Amo E.M., Urtti A. (2008). Current and future ophthalmic drug delivery systems. A shift to the posterior segment. Drug Discov. Today.

[24] Frenkel R.E., Haji S.A., La M., Frenkel M.P., Reyes A. (2010). A protocol for the retina surgeon’s safe initial intravitreal injections. Clin. Ophthalmol..

[25] Ausayakhun S., Yuvaves P., Ngamtiphakom S., Prasitsilp J. (2005). Treatment of cytomegalovirus retinitis in AIDS patients with intravitreal ganciclovir. J. Med. Assoc. Thai.

[26] Zhu Y., Zhu L., Wang X., Jin H. (2022). RNA-based therapeutics: An overview and prospectus. Cell Death Dis..

[27] Dowdy S.F. (2017). Overcoming cellular barriers for RNA therapeutics. Nat. Biotechnol..

[28] Crooke S.T., Witztum J.L., Bennett C.F., Baker B.F. (2018). RNA-targeted therapeutics. Cell Metab..

[29] Burnett J.C., Rossi J.J. (2012). RNA-based therapeutics: Current progress and future prospects. Chem. Biol..

[30] Alshaikh R.A., Waeber C., Ryan K.B. (2022). Polymer based sustained drug delivery to the ocular posterior segment: Barriers and future opportunities for the treatment of neovascular pathologies. Adv. Drug Deliv. Rev..

[31] Mainardes R.M., Urban M.C., Cinto P.O., Khalil N.M., Chaud M.V., Evangelista R.C., Gremiao M.P. (2005). Colloidal carriers for ophthalmic drug delivery. Curr. Drug Targets.

[32] Motwani S.K., Chopra S., Talegaonkar S., Kohli K., Ahmad F.J., Khar R.K. (2008). Chitosan-sodium alginate nanoparticles as submicroscopic reservoirs for ocular delivery: Formulation, optimisation and in vitro characterisation. Eur. J. Pharm. Biopharm..

[33] Pepic I., Hafner A., Lovric J., Pirkic B., Filipovic-Grcic J. (2010). A nonionic surfactant/chitosan micelle system in an innovative eye drop formulation. J. Pharm. Sci..

[34] Xu J., Wang Y., Li Y., Yang X., Zhang P., Hou H., Shi Y., Song C. (2007). Inhibitory efficacy of intravitreal dexamethasone acetate-loaded PLGA nanoparticles on choroidal neovascularization in a laser-induced rat model. J. Ocul. Pharmacol. Ther..

[35] Zhang X.P., Sun J.G., Yao J., Shan K., Liu B.H., Yao M.D., Ge H.M., Jiang Q., Zhao C., Yan B. (2018). Effect of nanoencapsulation using poly (lactide-co-glycolide) (PLGA) on anti-angiogenic activity of bevacizumab for ocular angiogenesis therapy. Biomed. Pharmacother..

[36] Seyfoddin A., Shaw J., Al-Kassas R. (2010). Solid lipid nanoparticles for ocular drug delivery. Drug Deliv..

[37] Souto E.B., Doktorovova S., Gonzalez-Mira E., Egea M.A., Garcia M.L. (2010). Feasibility of lipid nanoparticles for ocular delivery of anti-inflammatory drugs. Curr. Eye Res..

[38] Tong Y.C., Chang S.F., Kao W.W., Liu C.Y., Liaw J. (2010). Polymeric micelle gene delivery of bcl-xL via eye drop reduced corneal apoptosis following epithelial debridement. J. Control. Release.

[39] Bochot A., Fattal E. (2012). Liposomes for intravitreal drug delivery: A state of the art. J. Control. Release.

[40] Kaur I.P., Rana C., Singh H. (2008). Development of effective ocular preparations of antifungal agents. J. Ocul. Pharmacol. Ther..

[41] Kim J.H., Kim M.H., Jo D.H., Yu Y.S., Lee T.G., Kim J.H. (2011). The inhibition of retinal neovascularization by gold nanoparticles via suppression of VEGFR-2 activation. Biomaterials.

[42] Masse F., Ouellette M., Lamoureux G., Boisselier E. (2019). Gold nanoparticles in ophthalmology. Med. Res. Rev..

[43] Unnikrishnan G., Joy A., Megha M., Kolanthai E., Senthilkumar M. (2023). Exploration of inorganic nanoparticles for revolutionary drug delivery applications: A critical review. Discov. Nano.

[44] Tian Y., Zhang T., Li J., Tao Y. (2023). Advances in development of exosomes for ophthalmic therapeutics. Adv. Drug Deliv. Rev..

[45] Feng L., Li S.K., Liu H., Liu C.Y., LaSance K., Haque F., Shu D., Guo P. (2014). Ocular delivery of pRNA nanoparticles: Distribution and clearance after subconjunctival injection. Pharm. Res..

[46] Zhong C., Shi Z., Binzel D.W., Jin K., Li X., Guo P., Li S.K. (2024). Posterior eye delivery of angiogenesis-inhibiting RNA nanoparticles via subconjunctival injection. Int. J. Pharm..

[47] Fattal E., Bochot A. (2006). Ocular delivery of nucleic acids: Antisense oligonucleotides, aptamers and siRNA. Adv. Drug Deliv. Rev..

[48] Zhang Y., Shi Y., Khan M.M., Xiao F., Chen W., Tao W., Yao K., Kong N. (2024). Ocular RNA nanomedicine: Engineered delivery nanoplatforms in treating eye diseases. Trends Biotechnol..

[49] Xiao Y., Tang Z., Huang X., Chen W., Zhou J., Liu H., Liu C., Kong N., Tao W. (2022). Emerging mRNA technologies: Delivery strategies and biomedical applications. Chem. Soc. Rev..

[50] Saiding Q., Zhang Z., Chen S., Xiao F., Chen Y., Li Y., Zhen X., Khan M.M., Chen W., Koo S. (2023). Nano-bio interactions in mRNA nanomedicine: Challenges and opportunities for targeted mRNA delivery. Adv. Drug Deliv. Rev..

[51] Chaharband F., Daftarian N., Kanavi M.R., Varshochian R., Hajiramezanali M., Norouzi P., Arefian E., Atyabi F., Dinarvand R. (2020). Trimethyl chitosan-hyaluronic acid nano-polyplexes for intravitreal VEGFR-2 siRNA delivery: Formulation and in vivo efficacy evaluation. Nanomedicine.

[52] Alanazi J.S., Alqahtani F.Y., Aleanizy F.S., Radwan A.A., Bari A., Alqahtani Q.H., Abdelhady H.G., Alsarra I. (2022). MicroRNA-539-5p-loaded PLGA nanoparticles grafted with iRGD as a targeting treatment for choroidal neovascularization. Pharmaceutics.

[53] Tan G., Liu D., Zhu R., Pan H., Li J., Pan W. (2021). A core-shell nanoplatform as a nonviral vector for targeted delivery of genes to the retina. Acta Biomater..

[54] Wilson B., Geetha K.M. (2022). Lipid nanoparticles in the development of mRNA vaccines for COVID-19. J. Drug Deliv. Sci. Technol..

[55] Hou X., Zaks T., Langer R., Dong Y. (2021). Lipid nanoparticles for mRNA delivery. Nat. Rev. Mater..

[56] Tenchov R., Bird R., Curtze A.E., Zhou Q. (2021). Lipid nanoparticles–From liposomes to mRNA vaccine delivery, a landscape of research diversity and advancement. ACS Nano.

[57] Patel S., Ryals R.C., Weller K.K., Pennesi M.E., Sahay G. (2019). Lipid nanoparticles for delivery of messenger RNA to the back of the eye. J. Control. Release.

[58] Hagigit T., Abdulrazik M., Valamanesh F., Behar-Cohen F., Benita S. (2012). Ocular antisense oligonucleotide delivery by cationic nanoemulsion for improved treatment of ocular neovascularization: An in-vivo study in rats and mice. J. Control. Release.

[59] Mitra M., Kandalam M., Rangasamy J., Shankar B., Maheswari U.K., Swaminathan S., Krishnakumar S. (2013). Novel epithelial cell adhesion molecule antibody conjugated polyethyleneimine-capped gold nanoparticles for enhanced and targeted small interfering RNA delivery to retinoblastoma cells. Mol. Vis..

[60] Wang Y., Shahi P.K., Wang X., Xie R., Zhao Y., Wu M., Roge S., Pattnaik B.R., Gong S. (2021). In vivo targeted delivery of nucleic acids and CRISPR genome editors enabled by GSH-responsive silica nanoparticles. J. Control. Release.

[61] Wang Y., Shahi P.K., Xie R., Zhang H., Abdeen A.A., Yodsanit N., Ma Z., Saha K., Pattnaik B.R., Gong S. (2020). A pH-responsive silica-metal-organic framework hybrid nanoparticle for the delivery of hydrophilic drugs, nucleic acids, and CRISPR-Cas9 genome-editing machineries. J. Control. Release.

[62] Zhou T., He C., Lai P., Yang Z., Liu Y., Xu H., Lin X., Ni B., Ju R., Yi W. (2022). miR-204-containing exosomes ameliorate GVHD-associated dry eye disease. Sci. Adv..

[63] Zhao W., He X., Liu R., Ruan Q. (2023). Accelerating corneal wound healing using exosome-mediated targeting of NF-κB c-Rel. Inflamm. Regen..

[64] Tian Y., Zhang F., Qiu Y., Wang S., Li F., Zhao J., Pan C., Tao Y., Yu D., Wei W. (2021). Reduction of choroidal neovascularization via cleavable VEGF antibodies conjugated to exosomes derived from regulatory T cells. Nat. Biomed. Eng..

[65] Bao H., Tian Y., Wang H., Ye T., Wang S., Zhao J., Qiu Y., Li J., Pan C., Ma G. (2024). Exosome-loaded degradable polymeric microcapsules for the treatment of vitreoretinal diseases. Nat. Biomed. Eng..

[66] Fischer M.D., Ochakovski G.A., Beier B., Seitz I.P., Vaheb Y., Kortuem C., Reichel F.F.L., Kuehlewein L., Kahle N.A., Peters T. (2019). Efficacy and safety of retinal gene therapy using adeno-associated virus vector for patients with choroideremia: A randomized clinical trial. JAMA Ophthalmol..

[67] Ghazi N.G., Abboud E.B., Nowilaty S.R., Alkuraya H., Alhommadi A., Cai H., Hou R., Deng W.T., Boye S.L., Almaghamsi A. (2016). Treatment of retinitis pigmentosa due to MERTK mutations by ocular subretinal injection of adeno-associated virus gene vector: Results of a phase I trial. Hum. Genet..

[68] Binzel D.W., Khisamutdinov E.F., Guo P. (2014). Entropy-driven one-step formation of Phi29 pRNA 3WJ from three RNA fragments. Biochemistry.

[69] Binzel D.W., Li X., Burns N., Khan E., Lee W.J., Chen L.C., Ellipilli S., Miles W., Ho Y.S., Guo P. (2021). Thermostability, tunability, and tenacity of RNA as rubbery anionic polymeric materials in nanotechnology and nanomedicine-specific cancer targeting with undetectable toxicity. Chem. Rev..

[70] Shu Y., Pi F., Sharma A., Rajabi M., Haque F., Shu D., Leggas M., Evers B.M., Guo P. (2014). Stable RNA nanoparticles as potential new generation drugs for cancer therapy. Adv. Drug Deliv. Rev..

[71] Shi Z., Li S.K., Charoenputtakun P., Liu C.Y., Jasinski D., Guo P. (2018). RNA nanoparticle distribution and clearance in the eye after subconjunctival injection with and without thermosensitive hydrogels. J. Control. Release.

[72] Hu B., Zhong L., Weng Y., Peng L., Huang Y., Zhao Y., Liang X.J. (2020). Therapeutic siRNA: State of the art. Signal Transduct. Target. Ther..

[73] Chen X.L., Bai Y.J., Hu Q.R., Li S.S., Huang L.Z., Li X.X. (2016). Small interfering RNA targeted to ASPP2 promotes progression of experimental proliferative vitreoretinopathy. Mediat. Inflamm..

[74] Manavski Y., Carmona G., Bennewitz K., Tang Z., Zhang F., Sakurai A., Zeiher A.M., Gutkind J.S., Li X., Kroll J. (2014). Brag2 differentially regulates β1- and β3-integrin-dependent adhesion in endothelial cells and is involved in developmental and pathological angiogenesis. Basic Res. Cardiol..

[75] Zhang B., Hu Y., Ma J.X. (2009). Anti-inflammatory and antioxidant effects of SERPINA3K in the retina. Investig. Ophthalmol. Vis. Sci..

[76] Zhu W., Gui X., Zhou Y., Gao X., Zhang R., Li Q., Zhang H., Zhao J., Cui X., Gao G. (2024). Aurora kinase B disruption suppresses pathological retinal angiogenesis by affecting cell cycle progression. Exp. Eye Res..

[77] Song H.B., Jun H.O., Kim J.H., Fruttiger M., Kim J.H. (2015). Suppression of transient receptor potential canonical channel 4 inhibits vascular endothelial growth factor-induced retinal neovascularization. Cell Calcium.

[78] Kong Y.C., Sun B., Zhao K.X., Han M., Wang Y.C. (2013). Small interference RNA targeting vascular endothelial growth factor gene effectively attenuates retinal neovascularization in mice model. Chin. Med. J..

[79] Dong X., Wang Y.S., Dou G.R., Hou H.Y., Shi Y.Y., Zhang R., Ma K., Wu L., Yao L.B., Cai Y. (2011). Influence of Dll4 via HIF-1α-VEGF signaling on the angiogenesis of choroidal neovascularization under hypoxic conditions. PLoS ONE.

[80] Hirano Y., Sakurai E., Matsubara A., Ogura Y. (2010). Suppression of ICAM-1 in retinal and choroidal endothelial cells by plasmid small-interfering RNAs in vivo. Investig. Ophthalmol. Vis. Sci..

[81] Wu L., Guo F., Wu Y., Wang Q., Ma X., Zhao Y., Qin G. (2017). The role of FoxO1 in interleukin-1beta-induced autostimulation in retina endothelial cells and retinas of diabetic rats. Microvasc. Res..

[82] Tian X.F., Xia X.B., Xu H.Z., Xiong S.Q., Jiang J. (2012). Caveolin-1 expression regulates blood-retinal barrier permeability and retinal neovascularization in oxygen-induced retinopathy. Clin. Exp. Ophthalmol..

[83] Shang R., Lee S., Senavirathne G., Lai E.C. (2023). microRNAs in action: Biogenesis, function and regulation. Nat. Rev. Genet..

[84] Knott S.R.V., Maceli A., Erard N., Chang K., Marran K., Zhou X., Gordon A., Demerdash O.E., Wagenblast E., Kim S. (2014). A computational algorithm to predict shRNA potency. Mol. Cell.

[85] Feng Y., Wang J., Yuan Y., Zhang X., Shen M., Yuan F. (2018). miR-539-5p inhibits experimental choroidal neovascularization by targeting CXCR7. FASEB J..

[86] Hou H., Gao F., Liang H., Lv Y., Li M., Yao L., Zhang J., Dou G., Wang Y. (2018). MicroRNA-188-5p regulates contribution of bone marrow-derived cells to choroidal neovascularization development by targeting MMP-2/13. Exp. Eye Res..

[87] Mortuza R., Feng B., Chakrabarti S. (2014). miR-195 regulates SIRT1-mediated changes in diabetic retinopathy. Diabetologia.

[88] Zhao S., Li T., Li J., Lu Q., Han C., Wang N., Qiu Q., Cao H., Xu X., Chen H. (2016). miR-23b-3p induces the cellular metabolic memory of high glucose in diabetic retinopathy through a SIRT1-dependent signalling pathway. Diabetologia.

[89] Feng B., Chen S., McArthur K., Wu Y., Sen S., Ding Q., Feldman R.D., Chakrabarti S. (2011). miR-146a-mediated extracellular matrix protein production in chronic diabetes complications. Diabetes.

[90] Wang Y., Wang Y., Wang X., Ma Y., Li Z., Di Y. (2022). LncRNA TUG1 promotes apoptosis, invasion, and angiogenesis of retinal endothelial cells in retinopathy of prematurity via MiR-145-5p. Front. Med..

[91] Gutsaeva D.R., Thounaojam M., Rajpurohit S., Powell F.L., Martin P.M., Goei S., Duncan M., Bartoli M. (2017). STAT3-mediated activation of miR-21 is involved in down-regulation of TIMP3 and neovascularization in the ischemic retina. Oncotarget.

[92] Cui B., Sun J.H., Xiang F.F., Liu L., Li W.J. (2012). Aquaporin 4 knockdown exacerbates streptozotocin-induced diabetic retinopathy through aggravating inflammatory response. Exp. Eye Res..

[93] Zhang X.Z., Huang X., Qiao J.H., Zhang J.J., Zhang M.X. (2011). Inhibition of hypoxia-induced retinal neovascularization in mice with short hairpin RNA targeting Rac1, possibly via blockading redox signaling. Exp. Eye Res..

[94] Rezazadeh-Gavgani E., Oladghaffari M., Bahramian S., Majidazar R., Dolati S. (2023). MicroRNA-21: A critical underestimated molecule in diabetic retinopathy. Gene.

[95] Garba A.O., Mousa S.A. (2010). Bevasiranib for the treatment of wet, age-related macular degeneration. Ophthalmol. Eye Dis..

[96] Kaiser P.K., Symons R.C., Shah S.M., Quinlan E.J., Tabandeh H., Do D.V., Reisen G., Lockridge J.A., Short B., Guerciolini R. (2010). RNAi-based treatment for neovascular age-related macular degeneration by Sirna-027. Am. J. Ophthalmol..

[97] Shen J., Samul R., Silva R.L., Akiyama H., Liu H., Saishin Y., Hackett S.F., Zinnen S., Kossen K., Fosnaugh K. (2006). Suppression of ocular neovascularization with siRNA targeting VEGF receptor 1. Gene Ther..

[98] Nguyen Q.D., Schachar R.A., Nduaka C.I., Sperling M., Basile A.S., Klamerus K.J., Chi-Burris K., Yan E., Paggiarino D.A., Rosenblatt I. (2012). Dose-ranging evaluation of intravitreal siRNA PF-04523655 for diabetic macular edema (the DEGAS study). Investig. Ophthalmol. Vis. Sci..

[99] Nguyen Q.D., Schachar R.A., Nduaka C.I., Sperling M., Klamerus K.J., Chi-Burris K., Yan E., Paggiarino D.A., Rosenblatt I., Aitchison R. (2012). Evaluation of the siRNA PF-04523655 versus ranibizumab for the treatment of neovascular age-related macular degeneration (MONET Study). Ophthalmology.

[100] Pfeiffer N., Voykov B., Renieri G., Bell K., Richter P., Weigel M., Thieme H., Wilhelm B., Lorenz K., Feindor M. (2017). First-in-human phase I study of ISTH0036, an antisense oligonucleotide selectively targeting transforming growth factor beta 2 (TGF-β2), in subjects with open-angle glaucoma undergoing glaucoma filtration surgery. PLoS ONE.

[101] Xia X.B., Xiong S.Q., Song W.T., Luo J., Wang Y.K., Zhou R.R. (2008). Inhibition of retinal neovascularization by siRNA targeting VEGF(165). Mol. Vis..

[102] Gao Q., Wang W., Lan Y., Chen X., Yang W., Yuan Y., Tan J., Zong Y., Jiang Z. (2013). The inhibitory effect of small interference RNA protein kinase C-alpha on the experimental proliferative vitreoretinopathy induced by dispase in mice. Int. J. Nanomed..

[103] Hou Y., Xing L., Fu S., Zhang X., Liu J., Liu H., Lv B., Cui H. (2009). Down-regulation of inducible co-stimulator (ICOS) by intravitreal injection of small interfering RNA (siRNA) plasmid suppresses ongoing experimental autoimmune uveoretinitis in rats. Graefes Arch. Clin. Exp. Ophthalmol..

[104] Morgan-Warren P.J., O’Neill J., de Cogan F., Spivak I., Ashush H., Kalinski H., Ahmed Z., Berry M., Feinstein E., Scott R.A. (2016). siRNA-mediated knockdown of the mTOR inhibitor RTP801 promotes retinal ganglion cell survival and axon elongation by direct and indirect mechanisms. Investig. Ophthalmol. Vis. Sci..

[105] Antoszyk A., Katz B., Singh R., Gurses-Ozden R., Erlich S., Rothenstein D., Sharon N., Hodge J., Levin L., Miller N. (2013). A Phase I open label, dose escalation trial of QPI-1007 delivered by a single intravitreal (IVT) injection to subjects with low visual acuity and acute non-arteritic anterior ischemic optic neuropathy (NAION). Investig. Ophthalmol. Vis. Sci..

[106] AdisInsight QPI 1007. https://adisinsight.springer.com/drugs/800028983.

[107] Moreno-Montanes J., Sadaba B., Ruz V., Gomez-Guiu A., Zarranz J., Gonzalez M.V., Paneda C., Jimenez A.I. (2014). Phase I clinical trial of SYL040012, a small interfering RNA targeting β-adrenergic receptor 2, for lowering intraocular pressure. Mol. Ther..

[108] Benitez-Del-Castillo J.M., Moreno-Montanes J., Jimenez-Alfaro I., Munoz-Negrete F.J., Turman K., Palumaa K., Sadaba B., Gonzalez M.V., Ruz V., Vargas B. (2016). Safety and efficacy clinical trials for SYL1001, a novel short interfering RNA for the treatment of dry eye disease. Investig. Ophthalmol. Vis. Sci..

[109] GER-GROUP AGN-745 (Sirna-027)—Wet AMD Development Was Halted. https://amdbook.org/content/agn-745-sirna-027-wet-amd-development-was-halted.

[110] ScienceDirect Sirna 027. https://www.sciencedirect.com/topics/pharmacology-toxicology-and-pharmaceutical-science/sirna-027.

[111] AdisInsight PF4523655. https://adisinsight.springer.com/drugs/800025090.

[112] Isarna-Therapeutics Looking Beyond the Standard of Care for Eye Diseases. https://www.nature.com/articles/d43747-021-00054-6.

[113] Isarna-Therapeutics Phase I Dose Escalation Study to Investigate the Safety of ISTH0036 in Subjects with Glaucoma Undergoing Trabeculectomy. https://clinicaltrials.gov/study/NCT02406833.

[114] Ruckert R., Munk M.R., Wosikowski K., Janicot M. (2021). Long-term safety and efficacy of ISTH0036—A selective TGF-β2 blocking antisense oligonucleotide in preclinical and Phase 1 clinical studies. Investig. Ophthalmol. Vis. Sci..

[115] Quark-Pharmaceuticals Phase 2/3, Randomized, Double-Masked, Sham-Controlled Trial of QPI-1007 in Subjects with Acute Nonarteritic Anterior Ischemic Optic Neuropathy (NAION). https://clinicaltrials.gov/study/NCT02341560.

[116] Sylentis SYL040012, Treatment for Open Angle Glaucoma (SYLTAG). https://clinicaltrials.gov/study/NCT02250612.

[117] Sylentis Tivanisiran. https://sylentis.com/products-tivanisiran/.

[118] Sylentis Safety Study of Tivanisiran to Treat Dry Eye (FYDES). https://clinicaltrials.gov/study/NCT05310422.

[119] Mitra R.N., Nichols C.A., Guo J., Makkia R., Cooper M.J., Naash M.I., Han Z. (2016). Nanoparticle-mediated miR200-b delivery for the treatment of diabetic retinopathy. J. Control. Release.

[120] Du J., Zhao W., Wang Y., Cai Y. (2013). Lentivirus vector-mediated knockdown of erythropoietin-producing hepatocellular carcinoma receptors B4 inhibits laser-induced choroidal neovascularization. J. Ocul. Pharmacol. Ther..

[121] Li J., Li Y., Zhang M., Hu Z. (2012). Silencing of Rac1 expression via RNA interference inhibits retinal neovascularization in rats. Mol. Vis..

[122] Hu B., Zhang Y., Zeng Q., Han Q., Zhang L., Liu M., Li X. (2014). Intravitreal injection of ranibizumab and CTGF shRNA improves retinal gene expression and microvessel ultrastructure in a rodent model of diabetes. Int. J. Mol. Sci..

[123] Gold L., Ayers D., Bertino J., Bock C., Bock A., Brody E.N., Carter J., Dalby A.B., Eaton B.E., Fitzwater T. (2010). Aptamer-based multiplexed proteomic technology for biomarker discovery. PLoS ONE.

[124] Rohloff J.C., Gelinas A.D., Jarvis T.C., Ochsner U.A., Schneider D.J., Gold L., Janjic N. (2014). Nucleic acid ligands with protein-like side chains: Modified aptamers and their use as diagnostic and therapeutic agents. Mol. Ther. Nucleic Acids.

[125] Gragoudas E.S., Adamis A.P., Cunningham E.T., Feinsod M., Guyer D.R., the VEGF Inhibition Study in Ocular Neovascularization Clinical Trial Group (2004). Pegaptanib for neovascular age-related macular degeneration. N. Engl. J. Med..

[126] Tzoumas N., Riding G., Williams M.A., Steel D.H. (2023). Complement inhibitors for age-related macular degeneration. Cochrane Database Syst. Rev..

[127] Nakamura Y. (2021). Multiple therapeutic applications of RBM-007, an anti-FGF2 aptamer. Cells.

[128] Iturriaga-Goyon E., Vivanco-Rojas O., Magana-Guerrero F.S., Buentello-Volante B., Castro-Salas I., Aguayo-Flores J.E., Gracia-Mora I., Rivera-Huerta M., Sanchez-Bartes F., Garfias Y. (2021). AS1411 nucleolin-specific binding aptamers reduce pathological angiogenesis through inhibition of nucleolin phosphorylation. Int. J. Mol. Sci..

[129] Leaderer D., Cashman S.M., Kumar-Singh R. (2015). Topical application of a G-Quartet aptamer targeting nucleolin attenuates choroidal neovascularization in a model of age-related macular degeneration. Exp. Eye Res..

[130] Russell S.R., Drack A.V., Cideciyan A.V., Jacobson S.G., Leroy B.P., Van Cauwenbergh C., Ho A.C., Dumitrescu A.V., Han I.C., Martin M. (2022). Intravitreal antisense oligonucleotide sepofarsen in Leber congenital amaurosis type 10: A phase 1b/2 trial. Nat. Med..

[131] Adachi H., Hengesbach M., Yu Y.T., Morais P. (2021). From antisense RNA to RNA modification: Therapeutic potential of RNA-based technologies. Biomedicines.

[132] Dulla K., Slijkerman R., van Diepen H.C., Albert S., Dona M., Beumer W., Turunen J.J., Chan H.L., Schulkens I.A., Vorthoren L. (2021). Antisense oligonucleotide-based treatment of retinitis pigmentosa caused by USH2A exon 13 mutations. Mol. Ther..

[133] Zhang L., Li X., Zhao M., He P., Yu W., Dong J., Liu G., Li C., Shi X. (2006). Antisense oligonucleotide targeting c-fos mRNA limits retinal pigment epithelial cell proliferation: A key step in the progression of proliferative vitreoretinopathy. Exp. Eye Res..

[134] IVERIC-Bio Zimura in Participants with Geographic Atrophy Secondary to Dry Age-Related Macular Degeneration. https://clinicaltrials.gov/study/NCT02686658.

[135] Jaffe G.J., Westby K., Csaky K.G., Mones J., Pearlman J.A., Patel S.S., Joondeph B.C., Randolph J., Masonson H., Rezaei K.A. (2021). C5 Inhibitor avacincaptad pegol for geographic atrophy due to age-related macular degeneration: A randomized pivotal Phase 2/3 trial. Ophthalmology.

[136] Ribomic A Phase II Study of RBM-007 Alone and RBM-007 with Eylea® in Subjects with Wet Age-Related Macular Degeneration (TOFU). https://clinicaltrials.gov/study/NCT04200248.

[137] Ribomic Wet Age-Related Macular Degeneration (Wet AMD). https://www.ribomic.com/eng/pipeline/rbm007.php.

[138] Jaffe G.J., Ciulla T.A., Ciardella A.P., Devin F., Dugel P.U., Eandi C.M., Masonson H., Mones J., Pearlman J.A., Quaranta-El Maftouhi M. (2017). Dual antagonism of PDGF and VEGF in neovascular age-related macular degeneration: A Phase IIb, multicenter, randomized controlled trial. Ophthalmology.

[139] Ophthotech_Corporation A Phase 3 Safety and Efficacy Study of Fovista^®^ (E10030) Intravitreous Administration in Combination with Lucentis^®^ Compared to Lucentis^®^ Monotherapy. https://clinicaltrials.gov/study/NCT01944839.

[140] Bennett C.F. (2019). Therapeutic antisense oligonucleotides are coming of age. Annu. Rev. Med..

[141] Cideciyan A.V., Jacobson S.G., Drack A.V., Ho A.C., Charng J., Garafalo A.V., Roman A.J., Sumaroka A., Han I.C., Hochstedler M.D. (2019). Effect of an intravitreal antisense oligonucleotide on vision in Leber congenital amaurosis due to a photoreceptor cilium defect. Nat. Med..

[142] ProQR-Therapeutics A Study to Evaluate Efficacy, Safety, Tolerability and Exposure After a Repeat-Dose of Sepofarsen (QR-110) in LCA10 (ILLUMINATE). https://clinicaltrials.gov/study/NCT03913143.

[143] Murray S.F., Jazayeri A., Matthes M.T., Yasumura D., Yang H., Peralta R., Watt A., Freier S., Hung G., Adamson P.S. (2015). Allele-specific inhibition of rhodopsin with an antisense oligonucleotide slows photoreceptor cell degeneration. Investig. Ophthalmol. Vis. Sci..

[144] ProQR-Therapeutics A Study to Evaluate the Safety and Tolerability of QR-1123 in Subjects with Autosomal Dominant Retinitis Pigmentosa due to the P23H Mutation in the RHO Gene (AURORA). https://clinicaltrials.gov/study/NCT04123626.

[145] Diepen H.v., Dulla K., Chan H.L., Schulkens I., Beumer W., Vorthoren L., Besten C.d., Buil L., Turunen J., Miao J. (2019). QR-421a, an antisense oligonucleotide, for the treatment of retinitis pigmentosa due to USH2A exon 13 mutations. Investig. Ophthalmol. Vis. Sci..

[146] Laboratoires-Thea An Open-Label Extension Study to Evaluate Safety & Tolerability of QR-421a in Subjects with Retinitis Pigmentosa (HELIA). https://clinicaltrials.gov/study/NCT05085964.

[147] ProQR-Therapeutics Study to Evaluate Safety and Tolerability of QR-421a in Subjects with RP due to Mutations in Exon 13 of the USH2A Gene (Stellar). https://clinicaltrials.gov/study/NCT03780257.

[148] Khanani A.M., Boyer D.S., Wykoff C.C., Regillo C.D., Busbee B.G., Pieramici D., Danzig C.J., Joondeph B.C., Major J.C., Turpcu A. (2024). Safety and efficacy of ixoberogene soroparvovec in neovascular age-related macular degeneration in the United States (OPTIC): A prospective, two-year, multicentre phase 1 study. EClinicalMedicine.

[149] Campochiaro P.A., Avery R., Brown D.M., Heier J.S., Ho A.C., Huddleston S.M., Jaffe G.J., Khanani A.M., Pakola S., Pieramici D.J. (2024). Gene therapy for neovascular age-related macular degeneration by subretinal delivery of RGX-314: A phase 1/2a dose-escalation study. Lancet.

[150] MacLaren R.E., Groppe M., Barnard A.R., Cottriall C.L., Tolmachova T., Seymour L., Clark K.R., During M.J., Cremers F.P., Black G.C. (2014). Retinal gene therapy in patients with choroideremia: Initial findings from a phase 1/2 clinical trial. Lancet.

[151] OPKO-Health Safety and Efficacy Study of Small Interfering RNA Molecule (Cand5) to Treat Diabetic Macular Edema. https://clinicaltrials.gov/study/NCT00306904.

[152] OPKO-Health Safety and Efficacy Study of Small Interfering Ribonucleic Acid (RNA) Molecule (Cand5) to Treat Wet Age-Related Macular Degeneration. https://clinicaltrials.gov/study/NCT00259753.

[153] Allergan A Study Using Intravitreal Injections of a Small Interfering RNA in Patients with Age-Related Macular Degeneration. https://clinicaltrials.gov/study/NCT00395057.

[154] Quark-Pharmaceuticals PF-04523655 Dose Escalation Study, and Evaluation of PF-04523655 With/Without Ranibizumab in Diabetic Macular Edema (DME) (MATISSE). https://clinicaltrials.gov/study/NCT01445899.

[155] Sylentis Safety, Tolerability and Pharmacokinetic Profile of SYL1801 Eye Drops. https://clinicaltrials.gov/study/NCT04782271.

[156] HuidaGene-Therapeutics CRISPR/cas13-mediated RNA Targeting Therapy for the Treatment of Neovascular Age-Related Macular Degeneration Investigator-Initiated Trial (SIGHT-I). https://clinicaltrials.gov/study/NCT06031727.

[157] HuidaGene-Therapeutics Open-Label Dose-Escalation Study for CRISPR/cas13- RNA Targeting Therapy for the Treatment of Neovascular Age-Related Macular Degeneration in Phase I Trial (BRIGHT). https://clinicaltrials.gov/study/NCT06623279.

[158] Olix-Pharmaceuticals Evaluation of OLX10212 in Patients with Neovascular Age-Related Macular Degeneration. https://clinicaltrials.gov/study/NCT05643118.

